# KIT-dependent acute myeloid leukemias are responsive to LSD1 inhibition

**DOI:** 10.1186/s13148-026-02098-w

**Published:** 2026-05-14

**Authors:** Amal Kamal Abdel-Aziz, Mona Kamal Saadeldin

**Affiliations:** 1 Department of Pharmacology and Toxicology, Faculty of Pharmacy, Ain Shams University, Abbassia, Cairo, 11566 Egypt; 2 Department of Physiology and Pharmacology, College of Osteopathic Medicine, Sam Houston State University, Conroe, TX 77304 USA

**Keywords:** AML, KDM1A, KIT, Resistance, Sensitivity

## Abstract

Lysine specific demethylase 1 (LSD1 or KDM1A) is a FAD-dependent histone demethylase which is overexpressed in acute myeloid leukemias (AMLs). Indeed, several selective LSD1 inhibitors (LSD1is) are currently evaluated in clinical trials. We previously demonstrated that the mammalian target of rapamycin (mTOR) signalling is inhibited in LSD1i-sensitive AMLs and is activated in LSD1i-partially responsive/tolerant AMLs following LSD1i. Herein, we sought to systematically decipher the molecular basis underlying the responsiveness of AML cells to LSD1is. Genome-wide transcriptomic profiling, RT-qPCR and immunoblot analyses revealed that stem cell factor tyrosine kinase receptor (KIT) is preferentially overexpressed in LSD1i-sensitive AMLs compared to LSD1i-partially responsive/tolerant AMLs. Pharmacological and genetic interference with LSD1 repressed KIT transcription. c-Kit acted upstream of ERK/mTOR signalling. Chromatin immunoprecipitation studies revealed that LSD1 binds in proximity to KIT TSS and modulates repressive H3K9me2 marks. Inhibiting c-Kit augmented the anti-leukemic activity of LSD1is in KIT-dependent AML cells. Consistently, KIT mRNA positively correlated with LSD1 transcript levels in primary human AML biospecimens and pharmacological LSD1i repressed KIT transcription in primary human AML samples* ex vivo*. Collectively, this study sheds light on the epigenetic modulation of KIT transcription by LSD1 and underscores the therapeutic potential of co-targeting LSD1/KIT in KIT-dependent AMLs.

**Graphical Abstract:**

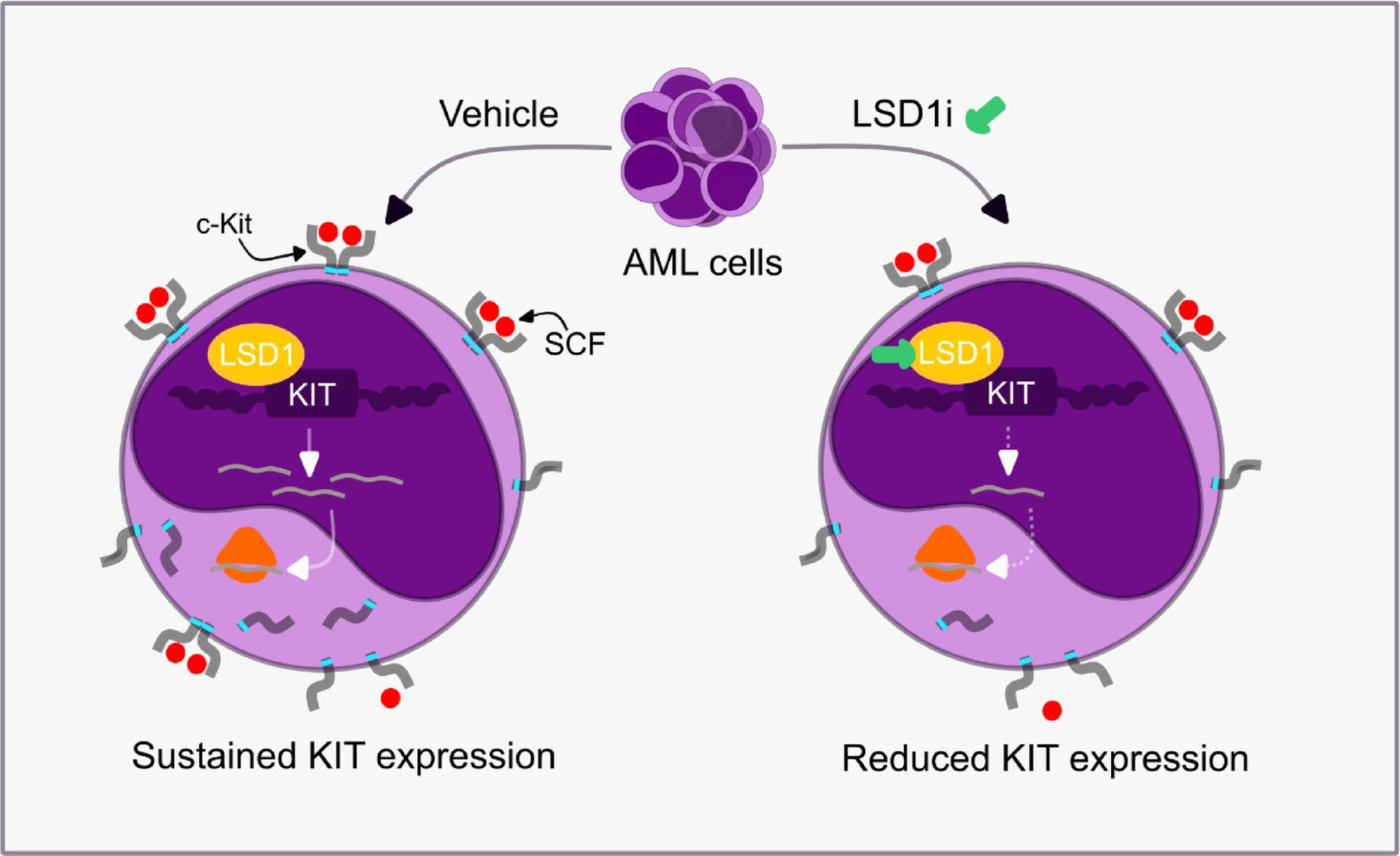

**Supplementary Information:**

The online version contains supplementary material available at 10.1186/s13148-026-02098-w.

## Highlights

- KIT-dependent AMLs are sensitive to LSD1 inhibition.
- LSD1 inhibition depresses KIT transcription.
- Forced KIT overexpression partially compromises the susceptibility of KIT-dependent AMLs to LSD1i.

**Supplementary Information:**

The online version contains supplementary material available at 10.1186/s13148-026-02098-w.

## Introduction

Lysine specific demethylase 1 (LSD1 or KDM1A) is a FAD-dependent chromatin modifier which has transcriptional co-repressor and co-activator capabilities [1–4]. Besides demethylating H3K4me1/2, H3K9me1/2 and H4K20me, LSD1 demethylates non-histone partners such as hypoxia inducible factor 1 alpha (HIF1α), p53 and forkhead box protein A 1 (FOXA1) [1, 2, 5–8]. Accumulating evidence demonstrated that the transcriptional modulatory effects of LSD1 are not necessarily linked to its canonical (demethylase) activity but rather its scaffolding role [9, 10].

Wada and colleagues have reported that LSD1 is highly expressed in most leukemic cell lines including that of acute myeloid leukemia (AML) but not normal human hematopoietic cells such as CD34^+^ hematopoietic stem and progenitor cells (HSPCs) [11]. Oncomine microarray database mining revealed that the mRNA levels of LSD1 are higher in primary human AML biospecimens compared to normal bone marrow cells [11]. Given their therapeutic potential, selective and potent LSD1 inhibitors (LSD1is) were developed and some are currently tested in clinical trials [12–16]. We and others previously reported that AML cells differentially respond to LSD1 inhibition [3, 17, 18]. Myeloid-lineage differentiation markers as CD86 was proposed as a surrogate biomarker for pharmacological LSD1is [19, 20]. Being a direct target of LSD1, CD86 was upregulated in both LSD1i- partially responsive (PR)/ irresponsive (IR) and LSD1i-completely responsive (CR) AML cells following their treatment with LSD1is [3]. We found that the mammalian target of rapamycin complex 1 (mTORC1) signalling was activated in LSD1i-PR/IR AML cells following LSD1 inhibition [3]. Digging deeper, LSD1i upregulated insulin receptor substrate 1 (IRS1) and triggered ERK1/2 signalling in LSD1i-PR/IR AML cells which acted upstream of mTORC1. Inhibiting IRS1/ERK/mTOR sensitized tolerant AML cells to LSD1i [3]. In harmony with our findings, Deb and colleagues also showed that co-targeting LSD1 and mTOR elicited superior anti-AML activity [21].

Within the context of LSD1i-CR AML cells, we found that LSD1i abrogated mTORC1 signalling [3]. However, the molecular basis underlying the responsiveness or vulnerability of LSD1i-CR AML is still unclear. Hence, the aim of this study was to systematically decipher the mechanisms underlying the sensitivity of LSD1i-CR AML cells to ultimately guide the clinicians towards rational stratification of AML patients for LSD1i therapy.

## Materials and methods

### Cell lines, primary AMLs and cell culture

Human AML cell lines were obtained from ATCC or DSMZ. UF1 (AML FAB M3), GDM-1 (AML FAB M4) and MOLM-13 (AML FAB M5a) cells were cultured in RPMI-1640 supplemented with 2mM L-glutamine, 20% fetal bovine serum (FBS) and 1% penicillin-streptomycin. HEL (AML FAB M6), HL-60 (AML FAB M2), KASUMI-1 (AML FAB M2), ML-2 (AML FAB M4), NB4 (AML FAB M3) and THP-1 (AML FAB M5) cells were cultured in RPMI-1640 supplemented with 2mM L-glutamine, 10% FBS and 1% penicillin-streptomycin. SKNO-1 (AML FAB M2) cells were cultured in RPMI-1640 media supplemented with 10% FBS, 2 mM L-glutamine and 10ng/ml GM-CSF. OCI-AML3 (AML FAB M4) cells were cultured in 80% α-MEM supplemented with 80% FBS, 2mM L-glutamine, and 1% penicillin-streptomycin. OCI-AML5 (AML FAB M4) cells were cultured in 80% alpha-MEM, 20% FBS, 2mM L-glutamine and 10ng/ml GM-CSF. M-07e (AML FAB M7) cells were supplemented with 80% Iscove’s MDM, 2mM L-glutamine, 20% FBS and 10ng/ml GM-CSF. Phoenix™ Ampho cells were cultured in DMEM supplemented with 2mM L-glutamine, 10% FBS and 1% penicillin-streptomycin. Primary murine c-Kit^+^ MLL-AF9 leukemic cells were a kind gift from Prof. Eric So. Cells were cultured in IMEM supplemented with 12.5% fetal calf serum, 12.5% horse serum, 2mM L-glutamine, 1% penicillin-streptomycin, hydrocortisone (10^− 6^M), amphotericin, 50mM β-mercaptoethanol, 20ng/ml interleukin-3, 20ng/ml interleukin-6, 100ng/ml stem cell factor (SCF). Cells were maintained in culture by the addition or replacement of fresh medium. Cells were maintained in a humidified tissue culture incubator at 37 °C with 5% CO_2_. All cells were tested for mycoplasma contamination before use.

### Reagents

DDP38003 was synthesized by Drug Discovery Unit, IFOM (Milan, Italy) as previously described [22]. ROCHE Complete™ EDTA-free Protease Inhibitor Cocktail (Catalog.No.11836170001), May-Grunwald, Giemsa, monoclonal anti-vinculin and anti-β-actin were purchased from Sigma Aldrich. Bio-Rad DC™ Protein Assay (Catalog.No.500 − 0114) was purchased from Biorad. Primary antibodies against c-Kit (D13A2) XP^®^ (3074), p-p70 S6 kinase (T389) (9234), total p70 S6K, p-4E-BP1 (T37/46) (2855), total 4E-BP1 (9644), p-pERK1/2 (T202/Y204) (4370) and total ERK1/2 (4695) were purchased from Cell Signalling Technology. APC mouse anti-human c-Kit (561118) was purchased from BD Pharmingen™.

### Retroviral and lentiviral constructs and transduction of AML cells

The hairpins used in this study are outlined in Table S1. Forty-eight and 72 h post-transfection, supernatants from transfected Ampho packaging cells were harvested and used for infection cycles. KASUMI-1 and THP-1 AML cells were transduced and selected as previously described [3]. For KIT knock down, TRIPZ inducible Lentiviral shRNA for Control and KIT (human) gene set were purchased from Dharmacon™ Reagents (Horizon Discovery).

### Cell proliferation and viability assays

To evaluate the effect on the proliferation and viability,AML cells were treated as indicated and cell counts were carried out using trypan blue (0.2%) exclusion and cell viability was assessed using Cell Titer-Glo™ Luminescent Cell Viability assay (Promega Corporation, USA) as previously described [3].

### Apoptosis detection using Annexin V/PI staining

AML cells were treated as indicated and the extent of apoptosis was assessed using Annexin V/ propidium iodide (PI) assay as previously described [17]. The percent of live (annexin V^−^, PI^−^), early apoptotic (annexin V^+^, PI^−^) and late apoptotic-necrotic (annexin V^+^, PI^+^) AML cells were analyzed using FlowJo software.

### Cell cycle analysis

AML cells were treated as indicated and cell cycle progression was assessed using PI as previously described [23]. Analysis of the cell cycle distribution pattern (G_0_/G_1_, S and G_2_/M) in AML cells was analyzed using FlowJo software.

### Real time-reverse transcriptase quantitative PCR (RT-qPCR) analysis

After AML treatment, cells were washed with ice-cold PBS and RNA was extracted using RNA extraction kit (Direct-zol™ RNA kit, Zymo research Corp). RT-qPCR was carried out as previously described [3]. The sequences of the RT-qPCR primers used in the present study are listed in Table S2.

### Western blot analysis

AML cells were seeded in T75 flasks and treated as indicated. Immunoblot analysis was carried out as described before [3]. Immunoreactive bands were detected using ECL chemiluminescent substrate (Amersham ECL Prime Western Blotting Detection Reagent, GE Healthcare life sciences).

### Cell surface c-Kit (CD117) staining and FACS analysis

Following their treatment, AML cells were harvested and centrifuged (3000 rpm for 10 min). Pellets were then resuspended in 100 µl 5% BSA in DPBS and incubated for 30 min. Cells were spun down 3000 rpm for 10 min and then resuspended with 50 µl primary APC mouse anti-human CD117 (c-Kit) diluted in 1% BSA in DBPS and incubated for 1 h at 4 °C. Cells were then washed twice with 200 µl 1% BSA in DPBS. Cells were resuspended with 100 µl ice-cold DPBS then 100 µl 2% formaldehyde in DPBS and incubated for 20 min on ice. Cells were then spun down and resuspended in 500 µl DPBS and FACS analysis was carried out using FACS Celesta. Data were analyzed using FlowJo software.

###  Chromatin immunoprecipitation quantitative PCR (ChIP-qPCR)

Chromatin immunoprecipitation (ChIP) assays for H3K9me2, H3K4me2, H3K27ac and IgG were performed as previously described [3]. After crosslinking with paraformaldehyde and quenching with glycine, AML cells were processed as previously described [3]. The sequences of the ChIP-qPCR primers used in this study are illustrated in Table S3.

### Bioinformatic analysis of RNA sequencing and ChIP sequencing

Raw count matrix of GSE125112 was downloaded from the NCBI Gene Expression Omnibus (GEO). Library size normalization and differential gene expression for OCI-AML3 versus KASUMI-1, KASUMI-1 (vehicle versus GSK2879552 [GSK-552]) and THP-1 (vehicle versus GSK-552) were carried out using DESeq2 algorithm [24, 25]. Genes were considered significantly differentially expressed (DEGs) at log2FC ≥ I 1 I and adjusted P value ≤ 0.05 threshold. R was used to run DESeq2 and to generate the volcano plots, heatmaps and other plots.

For ChIP-Seq, we exploited Cistrome platform, a web-based interactive visual analytics tool for exploring ChIP-Seq datasets [17, 26]. LSD1 ChIP-Seq of KASUMI-1 cells treated with either vehicle (GEO ID: GSM1844446), GSK-690 (selective reversible LSD1i) (GEO ID: GSM1844448) or RN-1 (selective irreversible LSD1i) (GEO ID: GSM1844447) and SKNO-1 cells (GEO ID: GSM1844440) were visualized and analyzed using UCSC Genome Browser. Likewise, H3K4me2 ChIP-Seq tracks of KASUMI1 cells treated with either vehicle (GEO ID: GSM1844449), GSK-690 (GEO ID: GSM1844451) or RN-1 (GEO ID: GSM1844450) were analyzed using UCSC data browser.

### Multi-omics analyses of AML cell lines

DepMap (Public 21Q2) Cancer Cell Line Encyclopedia (CCLE) platform was exploited to extract the gene expression (transcripts per million - TPM), copy number (relative ploidy), Sanger CRISPR Gene Effect (DepMap Public 25Q3 + Score, Chronos) and RPPA signal (log2) protein array data of AML cell lines [27].

### Co-expression analysis of LSD1 and KIT expression in primary biospecimens obtained from AML patients

Correlation analysis of LSD1 and KIT mRNA in AML biospecimens obtained from AML TCGA dataset (NEJM, 2013), Pediatric AML TARGET dataset (2018) and OHSU AML dataset (Cancer cell, 2022) was carried out using co-expression analysis tool of cBioportal online database (https://www.cbioportal.org/.). The coefficient and P value were assessed by Spearman’s and Pearson’s rank correlation analyses.

### Statistical analysis

Data analysis was performed using GraphPad Instat as outlined below; unless otherwise indicated, data are presented as mean ± standard deviation (SD). Student’s t-test was exploited to compare two groups. Multiple comparisons (> 2 treatment groups) were performed using One-way ANOVA followed by Bonferroni test for *post-hoc* analysis or two-way ANOVA followed by Bonferroni *post-hoc* test. Graphs were presented using Graphpad Prism and R softwares.

## Results

### LSD1i responsive AML cells express higher levels of stem cell growth factor receptor (c-Kit)

**Fig. 1:**
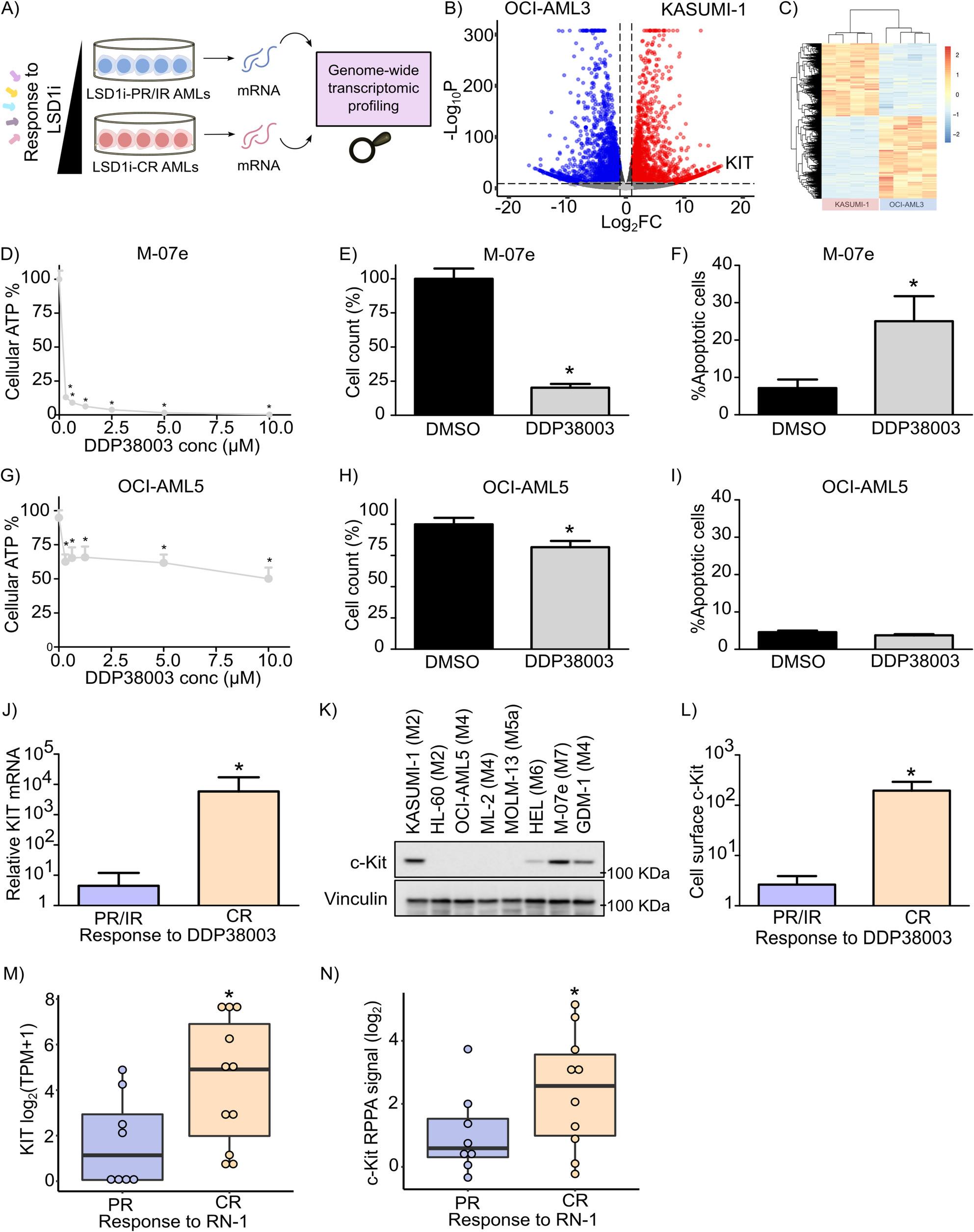
Stem cell factor tyrosine kinase receptor (c-Kit) is overexpressed in acute myeloid leukemia (AML) cells which are completely responsive (CR) to lysine specific demethylase 1 inhibition (LSD1i) compared to LSD1i-partially responsive (PR) or irresponsive (IR) AML cells. **A** Schematic representation of the workflow implemented in the genome wide transcriptome analysis of LSD1i-CR AML cells (as KASUMI-1) as well as LSD1i-PR/IR AML cells (as OCI-AML3). **B** Volcano plot depicting significantly differentially expressed genes (DEGs) in LSD1i-sensitive KASUMI-1 AML cells compared to LSD1i PR/IR OCI-AML3 AML cells. The cutoff of Log2FC =I 1 I and P value = 10e^-10^. **C** Heatmap representation of differentially expressed genes (DEGs) in KASUMI-1 compared to OCI-AML3 (FDR < 0.05 - Z-score values). **D** Percent of cellular ATP levels assessed using Cell Titer-Glo™ Luminescent Cell Viability assay of LSD1i-sensitive M-07e cells 144h following their treatment with the indicated concentrations of DDP38003 (0-10 μM) [IC50 = 0.183±0.01μM].*: P≤0.05 compared to vehicle treated cells assessed using One way ANOVA followed by Dunnett’s post-hoc test. **E** Percent of cell count of M-07e cells 7 days following their treatment with either DMSO or DDP38003 (0.5 μM).*: P≤0.05 compared to vehicle treated cells assessed using Student’s t test. **F** Percent of apoptotic cells of M-07e cells following their treatment with either vehicle (DMSO) or DDP38003 (0.5 μM) for 7 days.*: P≤0.05 compared tovehicle treated cells assessed using Student’s t test. **G** Percent of cellular ATP levels assessedusing Cell Titer-Glo™ Luminescent Cell Viability assay of LSD1i-PR/IR (OCI-AML5) 144hfollowing their treatment with the indicated concentrations of DDP38003 (0-10 μM) [IC>10μM].*: P≤0.05 compared to vehicle treated cells assessed using One way ANOVA followed by Dunnett's *post-hoc* test. **H** Percent of cell count of OCI-AML5 cells 7 days following theirtreatment with either DMSO or DDP38003 (0.5 μM).*: P≤0.05 compared to vehicle treated cells assessed using Student’s t test. **I** Percent of apoptotic cells of OCI-AML5 cells following their treatment with either vehicle (DMSO) or DDP38003 (0.5 μM) for 7 days. *: P≤0.05 compared tovehicle-treated cells assessed using Student’s t test **J** Relative KIT mRNA level in DDP38003-CR and DDP38003-PR/IR AML cells assessed using RT-qPCR. *: P≤0.05 compared to DDP38003-CR AML cells assessed using Student’s t test. **K** Immunoblot analysis of c-Kit in DDP38003-CR and DDP38003-PR/IR AML cells. Vinculin served as the loading control. **L** Quantitation of the mean fluorescence intensity (MFI) of the basal levels of cell surface c-Kit receptor in DDP38003-CR and DDP38003-PR/IR AML cells. *: P≤0.05 compared to DDP38003-CR AML cell lines assessed using Student’s t test. M-N) Box plot depicting the expression levels of KIT mRNA [log2(TPM+1)] **M** and c-Kit reverse protein phase array (RPPA) (log2) **N** obtained from Cancer Cell Line Encyclopedia (CCLE) of RN-1 CR and RN-1 PR AML cell lines (which are represented as dots). *: P≤0.05 compared to RN-1 CR AML cells.

We and others previously reported that AML cells exhibit heterogeneous responses to selective LSD1 inhibitors (LSD1is) including DDP38003, RN-1 and GSK2879552 (GSK-552) [3, 17, 18]. Herein, we sought to gain insights into the molecular mechanisms contributing to differential AML responsiveness to LSD1i [3, 17, 18]. To this end, we initially performed differential gene expression analysis of LSD1i-CR AML cells (such as KASUMI-1) and LSD1i-PR/IR AML cells (such as OCI-AML3) which we previously demonstrated their differential responses to pharmacological LSD1 inhibition and genetic LSD1 knock down [3] (Fig. 1A). Indeed, genome-wide transcriptomic analyses revealed that 8272 genes were differentially expressed (3526 upregulated and 4746 downregulated genes respectively) in KASUMI-1 compared to OCI-AML3 (Table S4). Notably, stem cell growth factor receptor (KIT) was among the top five highly expressed genes in LSD1i-CR KASUMI-1 cells when compared to LSD1i-PR/IR OCI-AML3 cells (Fig. 1B and C). c-Kit is a tyrosine kinase receptor which acts upstream of diverse signalling pathways including mTOR [28–30].

Next, we sought to examine whether such differential KIT levels were reproducible in other LSD1i-CR and LSD1i-PR/IR AML cells. Indeed, based on the responses of AML cells to DDP38003, we previously classified AML cells into two cohorts: LSD1i-CR AML cells (including KASUMI-1 (FAB: M2), SKNO-1 (FAB: M2) and UF1 (FAB: M3)) and LSD1i-PR/IR AML cells (as OCI-AML3 (FAB: M4), THP-1 (FAB: M5) and NB4 (FAB: M3)) [3]. Extending the panel of AML cell lines tested, herein we further evaluated the anti-leukemic activity of DDP38003 on HL-60 (FAB: M2), GDM-1 (FAB: M4), OCI-AML5 (FAB: M4), ML-2 (FAB: M4), MOLM-13 (FAB: M5a), HEL (FAB: M6) and M-07e (FAB: M7). As expected, we observed heterogeneous responses of AML cell lines to DDP38003 spanning from PR/IR (as OCI-AML5, ML-2 and MOLM-13) to CR (as M-07e, GDM-1 and HEL) (Fig. 1D-I and Supplementary Fig. 1A-1J). This is evidenced by significantly higher reduction in the viability and cell count as well as induction of apoptosis and G_0_/G_1_ cell cycle arrest in DDP38003-CR AML cell lines as compared to DDP38003-PR/IR AML cells (Fig. 1D-I and Supplementary Fig. 1A-1J). Consistently, RT-qPCR, immunoblot and flow cytometry analyses confirmed that DDP38003-CR AML cells express substantially higher levels - in logarithmic scale - of KIT mRNA and c-Kit protein when compared to DDP38003-PR/IR AML cells (Fig. 1J-L and Supplementary Fig. 2 A). Next, we questioned whether the high expression of KIT in DDP38003-CR AML cells is an observation restricted to DDP38003 and not other LSD1is. Hence, we took advantage of the study of Mcgrath and colleagues in which they evaluated the response of a panel of AML cell lines to RN-1, another selective irreversible LSD1 inhibitor [17]. According their vulnerability to RN-1, AML cells were stratified into two categories: (i) Sensitive (with 40–100% maximal growth inhibition) and (ii) Partial (with less than 70% decrement in cell number) [17]. We questioned whether AML cells expressing high levels of KIT are more vulnerable to RN-1. By further examining KIT expression (RNA sequencing), c-Kit reverse protein phase array (RPPA) and KIT copy number profiles of AML cell lines using the Cancer Cell Line Encyclopedia (CCLE) [31, 32], we observed that RN-1 sensitive AML cells expressed significantly higher levels of KIT transcript and c-Kit protein compared to RN-1 PR AML cells (Fig. 1M-N). Nonetheless, KIT copy number and the transcript levels of other tyrosine kinase receptors including platelet derived growth factor receptor β (PDGFRβ), AXL, mesenchymal-epithelial transition factor receptor (MET) and epidermal growth factor receptor (EGFR) were comparable in RN-1-CR AML cells and RN-1-PR AML cells (Supplementary Figure 2B-2F).

Smitherman and colleagues previously reported that the mean anti-proliferative EC50 of GSK-552, another selective and irreversible LSD1 inhibitor, on a panel of 20 AML cell lines is approximately 140 nM [18]. Setting an EC50 cut-off of approximately 100 nM, we categorized AML cell lines into: GSK-552 CR and GSK-552 PR/IR cohorts. Consistently, KIT mRNA and c-Kit protein levels were significantly higher in GSK-552 CR AML cells compared to GSK-552 PR/IR AML cells (Supplementary Figure 3A-3B). Nonetheless, the transcript levels of other tyrosine kinase receptors as AXL, EGFR and fms-like tyrosine kinase 3 (FLT3) did not differ in GSK-552 CR AML cells and GSK-552 PR/IR AML cells (Supplementary Figure 3C-3E). Altogether, the present findings indicate that LSD1i-CR AML cells express substantially higher levels of KIT compared to LSD1i-PR/IR AML cells.

### Pharmacological as well as genetic interference of LSD1 depresses KIT transcription

**Fig. 2:**
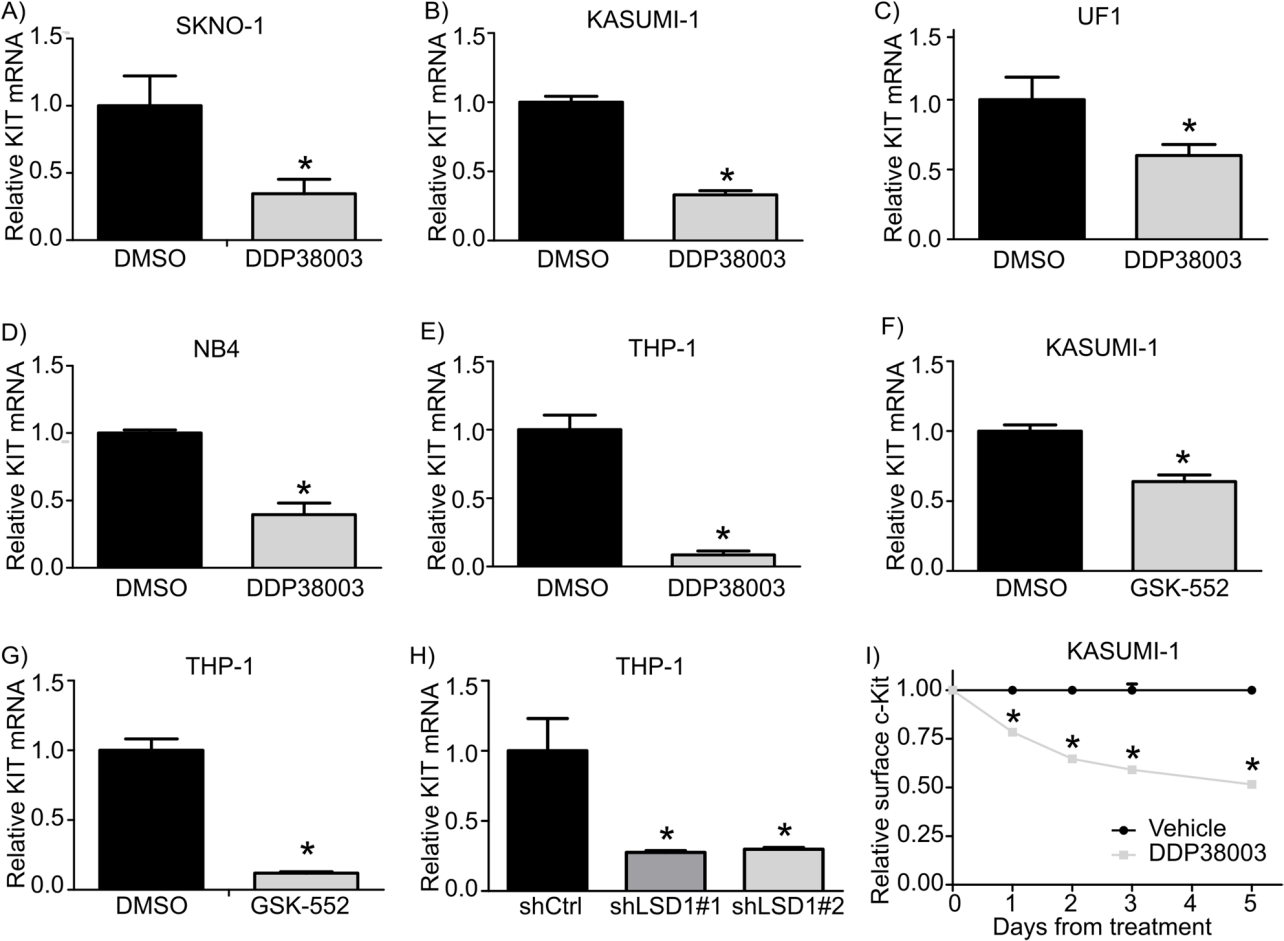
Lysine specific demethylase 1 inhibition (LSD1i) represses stem cell factor tyrosine kinase receptor (KIT) transcription in acute myeloid leukemia (AML) cells. **A**-**E** Effect of treatment with DDP38003 (0.5 µM) on KIT transcription in SKNO-1 (**A**), KASUMI-1 (**B**), UF1 (**C**), NB4 (**D**) and THP-1 (**E**) AML cells assessed using RT-qPCR. *: *P* ≤ 0.05 compared to vehicle treated AML cells using Student’s t test. **F**-**G**) Relative KIT transcript levels in KASUMI-1 (**F**) and THP-1 (**G**) following 4 days of their treatment with either vehicle or GSK2879552 (GSK-552) was assessed using RNA-Sequencing (GSE125112 dataset). *: *P* ≤ 0.05 compared to vehicle treated AML cells using Student’s t test. **H** Effect of knock down of LSD1 on KIT transcription analysed using RT-qPCR in THP-1 cells transduced with vectors expressing control (shCtrl) or shRNAs against LSD1. Data were statistically analyzed using One way ANOVA followed by Bonferroni *post-hoc* test, ^*^: *P* ≤ 0.05 compared to shCtrl transduced THP-1 cells. **I** Quantitative analysis of the relative mean fluorescence intensity reflecting cell surface expression level of c-Kit in KASUMI-1 cells following their treatment with either vehicle or DDP38003 (0.5 µM) for the indicated time points. Data were statistically analyzed using two-way ANOVA followed by Bonferroni *post-hoc* test^*^: *P* ≤ 0.05 compared to vehicle-treated cells.

Next, we sought to investigate whether LSD1 inhibition modulates KIT expression. Notably, two different pharmacological LSD1is including DDP38003 and GSK-552 significantly reduced KIT mRNA levels in LSD1i-CR and LSD1i-IR/PR AML cells (Fig. 2A-G). Next, we exploited LSD1-targeting small hairpins (shRNAs) to silence LSD1. We previously demonstrated that LSD1 knock down was tolerated in LSD1i-PR/IR AML cells such as THP-1 but sharply affected the proliferation of LSD1i-vulnerable AML cells such as KASUMI-1 resulting in the complete and partial counter-selection of one of the shLSD1 against the wild-type cells [3]. Consistently, LSD1 knock down significantly depressed KIT transcription in LSD1i-IR/PR and LSD1i-CR AML cells (Fig. 2H and Supplementary Figure 4 A).

DDP38003 also significantly compromised the proliferation and viability of murine c-Kit^+^ AML cells, promoted myeloid-lineage differentiation and abrogated KIT transcription (Supplementary Figure 4B-4G). Flow cytometric analysis confirmed that LSD1 inhibition significantly abrogated the cell surface levels of c-Kit receptor protein in DDP38003-CR AML cells such as KASUMI-1 (Fig. 2I). Consistently, inspecting the RPPA datasets developed by Curtiss and colleagues [33] revealed that 24 h monotherapy of KASUMI-1 cells with ORY-1001, a selective LSD1 inhibitor, significantly reduced the protein levels of c-Kit but not MET and AXL tyrosine kinase receptors (Supplementary Figure 4 H–4 J). Altogether, these findings highlight the negative modulatory effects of pharmacological and genetic LSD1i on KIT transcription in AML cells.

### LSD1 epigenetically modulates KIT transcription and erases repressive H3K9me2 marks in vicinity to the transcription start site of KIT

Lysine specific demethylase 1 (LSD1) exhibits context-dependent transcriptional co-repressor and co-activator capabilities [1, 2, 9, 34]. The latter was linked to LSD1-mediated demethylation of the H3K9me1/2 which are facultative heterochromatin marks [2, 35]. Given the downregulatory effects of LSD1i on KIT transcription, we asked: (i) whether LSD1 binds in proximity to the transcription start site (TSS) of KIT gene and, (ii) if yes, whether LSD1i modulates the repressive H3K9me2 marks in the vicinity of KIT TSS/promoter.

**Fig. 3:**
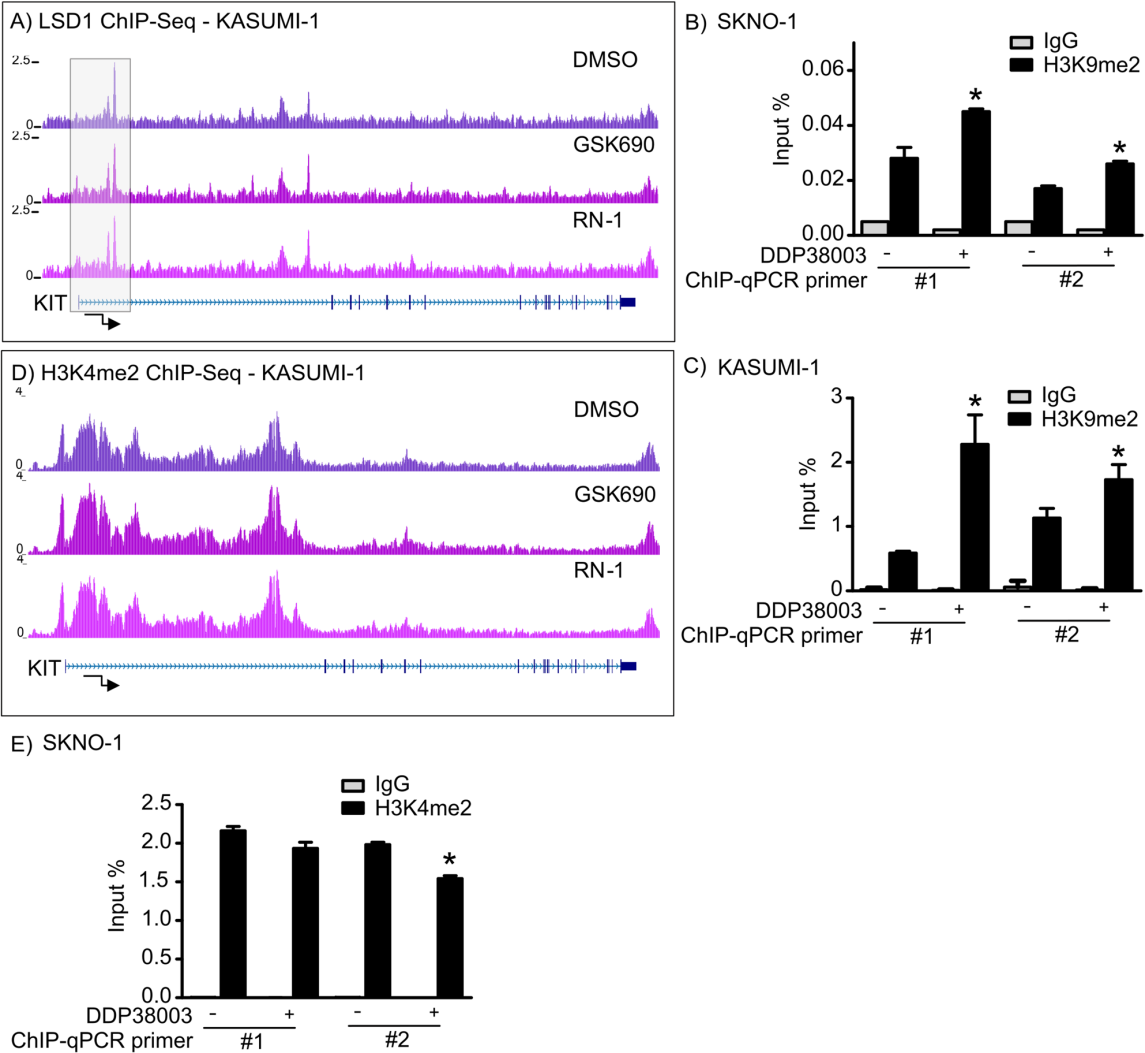
Lysine specific demethylase 1 (LSD1) epigenetically modulates KIT transcription and relieves repressive H3K9me2 marks in the vicinity of the transcription start site (TSS) of KIT gene in acute myeloid leukemia (AML) cells. A Cistrome LSD1 ChIP-Seq tracks depicting KIT TSS in KASUMI-1 cells treated with either DMSO, GSK-690 (selective reversible LSD1i) or RN-1 (selective irreversible LSD1i) respectively (McGrath et al. 2016 dataset). B-C H3K9me2 ChIP-qPCR analysis of SKNO-1 and KASUMI-1 cells treated with either vehicle (PEG) or DDP38003 (0.5 µM). Data were statistically analyzed using Student’s t test, *: P ≤ 0.05 compared to vehicle-treated cells. D Cistrome H3K4me2 ChIP-Seq tracks depicting KIT TSS in KASUMI-1 cells treated with either DMSO, GSK-690 or RN-1 respectively (McGrath et al. 2016 dataset). E H3K4me2 ChIP-qPCR analysis of SKNO-1 cells following their treatment with either vehicle (PEG) or DDP38003 (0.5 µM). Data were statistically analyzed using Student’s t test, *: P ≤ 0.05 compared to vehicle-treated cells.

Interrogating ENCODE and FANTOM5 identified LSD1 as one of the potential transcription factors binding to KIT enhancer. Cistrome LSD1 ChIP-seq and ChIP-qPCR analyses confirmed that LSD1 binds ~ 5 kb from the TSS of KIT gene in LSD1i-CR and LSD1i-PR/IR AML cells (Fig. 3A and Supplementary Figure 5 A-5 C). Notably, LSD1 inhibition increased the occupancy of H3K9 dimethylation and reduced H3K27 acetylation close to the TSS of KIT gene as compared to vehicle-treated AML cells (Fig. 3B-C and Supplementary Figure 5D-5E). Nonetheless, LSD1 inhibition was not associated with increment of H3K4 dimethylation at KIT TSS (Fig. 3D–E). Altogether, our data imply that LSD1 epigenetically modulates KIT transcription which is associated with H3K9me2 demethylation in AML cells.

### LSD1i-mediated suppression of KIT at least partially contributes to the anti-leukemic activity of LSD1i in KIT-dependent AML cells

**Fig. 4:**
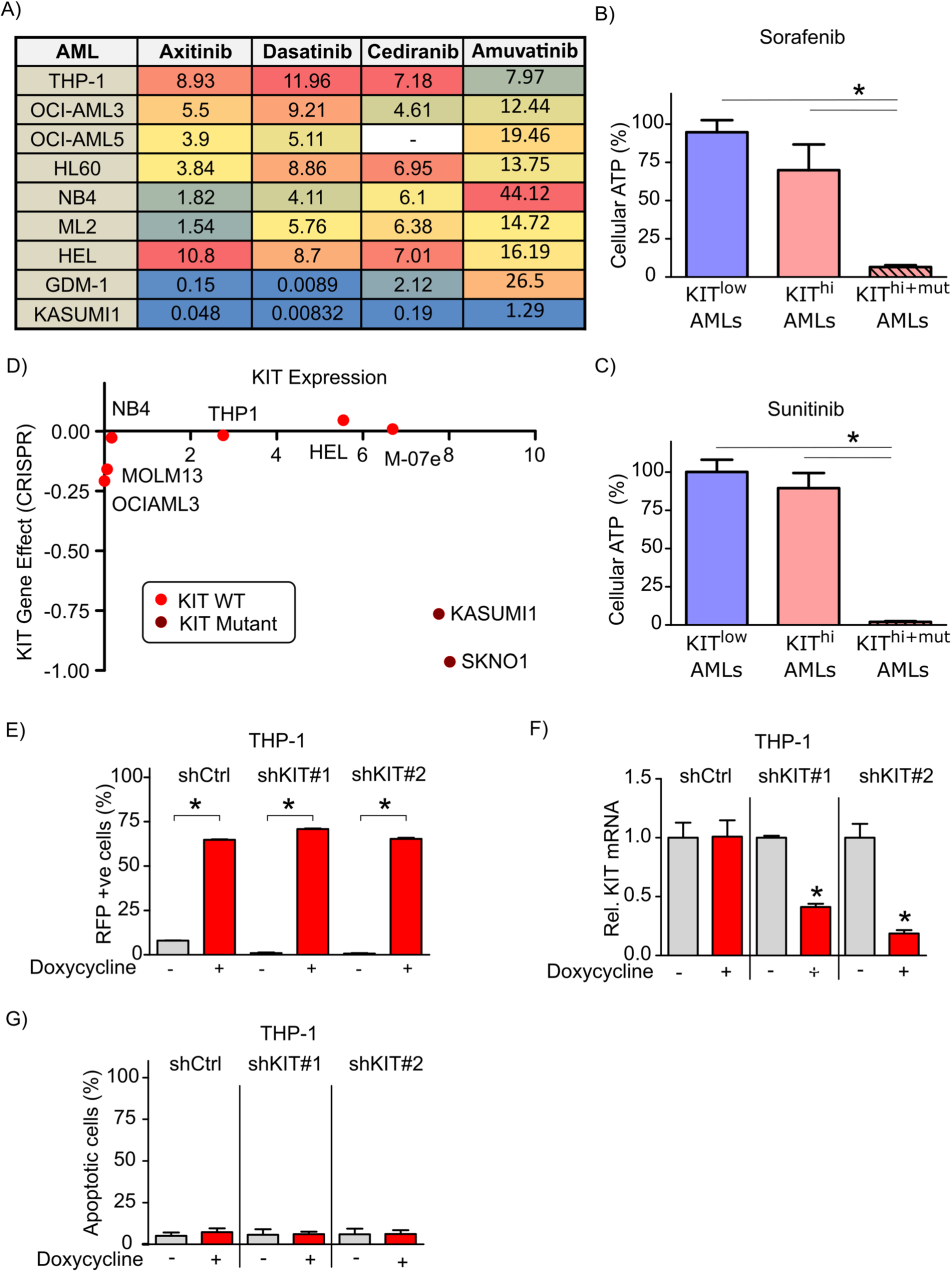
Lysine specific demethylase 1 inhibitor (LSD1i)-completely responsive (CR) KIT^hi+mutant^ AMLs are more dependent on KIT compared to LSD1i-CR KIT^hi^ AMLs and LSD1i-resistant KIT^low^ AMLs expressing wild type KIT. **A** The median inhibitory concentrations (IC50) of the indicated AML cells following their treatment with the following multityrosine kinase inhibitors (axitinib, dasatinib, cediranib and amuvatinib). The values of the IC50s were obtained from the Genomics of Drug Sensitivity in Cancer database (https://www.cancerrxgene.org). **B**-**C** The relative percent of cell viability assessed using ATP CellTiter Glo assay of DDP38003-partially responsive (PR)/irresponsive (IR) KIT^low^ AML cells expressing low levels of wild type KIT (THP-1, NB4, OCI-AML3, OCI-AML5, HL60 and ML-2), DDP38003-CR KIT^hi^ AML cells expressing high levels of wild type KIT (UF1, GDM-1, M0e-7 and HEL) and DDP38003-CR KIT^hi+mutant^ AML cells (KASUMI-1 and SKNO-1) expressing high levels of mutated KIT following their 144 h treatment with one of the following multikinase c-Kit inhibitors (500 nM): sorafenib (**B**) and sunitinib (**C**). Data were statistically analyzed using one-way ANOVA followed by Bonferroni post-hoc test. *: P ≤ 0.05 compared to vehicle-treated cells. **D** Dot plot depicting the gene dependency effect (CRISPR, DepMap Public 25Q3 + Score, Chronos) of AML cells on KIT plotted on the Y‑axis versus the corresponding mRNA expression level (log2(TPM + 1)) of KIT plotted on the X‑axis. Bright red dots correspond to wild type c-Kit expressing AML cell lines whereas dark red dots correspond to AML cell lines carrying mutated c-Kit which promotes constitutive activation of c-Kit signalling. **E** Percent of RFP positive (%) in stably transduced THP-1 cells with doxycycline-inducible lentiviral vectors expressing either: shCtrl, shKIT#1 or, shKIT#2 treated with either vehicle or doxycycline (1 µg/ml) assessed using FACS. *: P ≤ 0.05 compared to the corresponding vehicle-treated transduced THP-1 cells. **F**-**G** Relative KIT mRNA levels (**F**) assessed using RT-qPCR and percent of apoptotic cells (**G**) assessed using FACS of stably transduced THP-1 cells with doxycycline-inducible lentiviral vectors expressing either: shCtrl, shKIT#1 or, shKIT#2 treated with either vehicle or doxycycline (1 µg/ml). Data were statistically analyzed using Student’s t test, *: P ≤ 0.05 compared to the corresponding vehicle-treated transduced THP-1 cells

Based on the aforementioned results, we questioned the potential differential dependency of LSD1i-PR/IR and LSD1i-CR AML cells on KIT. To address this query, we first exploited the Genomics of Drug Sensitivity in Cancer (GDSC) database which comprises the median inhibitory concentrations (IC50) of a large panel of cancer cell lines including AMLs to diverse pharmacological agents including multityrosine kinase inhibitors (TKIs) which inhibits c-Kit such as axitinib (targets: PDGFR, KIT and VEGFR), dasatinib (targets: ABL, SRC, Ephrins, PDGFR and KIT), cediranib (targets: VEGFR, FLT1, FLT2, FLT3, FLT4, KIT and PDGFRB) and amuvatinib (targets: KIT, PDGFRA and FLT3) [36, 37]. Of note, LSD1i-CR KIT^hi^ AML such as KASUMI-1 cells which harbour a specific mutation in KIT which promotes its constitutive activation [38] displayed the highest vulnerability to pharmacological TKIs inhibitors when compared to LSD1i-CR KIT^hi^ AMLs and LSD1i-PR/IR KIT^low^ AML cells expressing wild type (WT) KIT (Fig. 4A). We further confirmed these findings using two other TKIs such as sorafenib and sunitinib which have potent inhibitory effects on c-Kit (Fig. 4B-C).

To exclude the potential off-target effects of pharmacological TKIs, we took advantage of the Cancer Dependency Map (DepMap) datasets of Sanger CRISPR Gene Effect (DepMap Public 25Q3 + Score, Chronos) to examine the gene effect of CRISPR-mediated genetic perturbation of KIT in AML cells [27, 39, 40]. Lower Chronos score reflected a higher probability that KIT is crucial for the survival of the inspected AML cell line. Notably, KIT^hi+mutant^ AML cells (such as KASUMI-1 and SKNO-1) - but not wild type KIT^hi^ expressing AMLs (such as M0-e7 and HEL) and wild type KIT^low^ expressing AML cells (such as THP-1, NB4, OCI-AML3 and MOLM13) - were vulnerable to CRISPR-Cas mediated KO of KIT (Fig. 4D).

Next, we transduced LSD1-CR KIT^hi+mutant^ AML cells such as KASUMI-1 and LSD1-PR/IR KIT^low^ AML cells such as THP-1 with pTRIPZ inducible lentiviral shRNA vectors against Control or KIT which include TurboRFP and shRNA as part of a single transcript permitting the visualization of shRNA-expressing cells. Consistently, KASUMI-1 cells were counter-selected upon doxycycline treatment confirming their dependency on KIT (data not shown). Conversely, doxycycline treatment significantly increased the percent of RFP positive cells confirming the expression of shRNAs against Control or KIT and reduced KIT transcription in shKIT transduced THP-1 cells which were tolerant to KIT KD (Fig. 4E-G). Thus, these findings suggested that LSD1i-CR KIT^hi+mutant^ AMLs – but not LSD1i-CR KIT^hi^ and LSD1i-PR/IR KIT^low^ AMLs might be dependent on KIT.

**Fig. 5:**
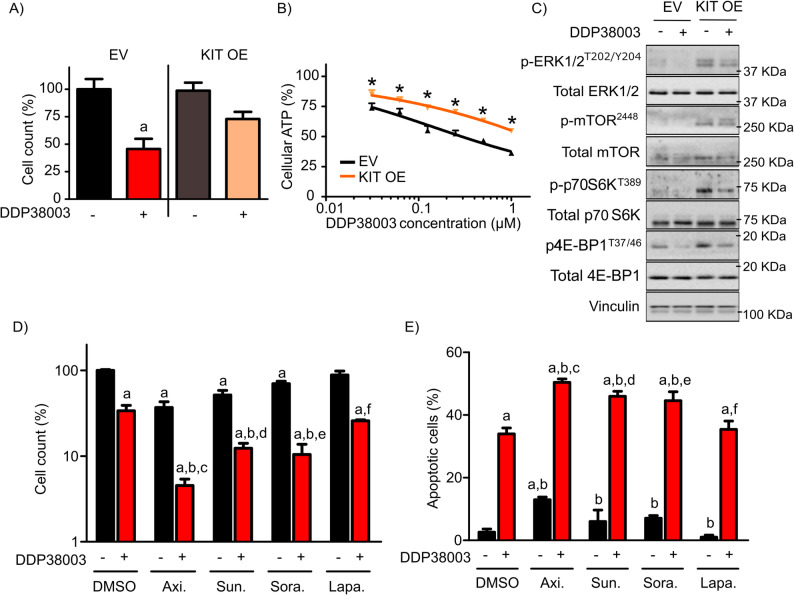
Modulating KIT plays a role – at least partly – in regulating mTOR signalling and mediating the antileukemic activity of LSD1i in sensitive AMLs. **A** Relative percent of cell count of KASUMI-1 cells transduced with empty vector (EV) or KIT treated with either vehicle or DDP38003 (0.5 µM). ^*^: *P* ≤ 0.05 compared to transduced KASUMI-1 cells expressing empty vector. **B** Cell viability (%) of transduced KASUMI-1 cells expressing empty vector (EV) or KIT was determined following their treatment with the indicated concentrations of DDP38003 using Cell Titer-Glo™ Luminescent Cell Viability assay. Data were statistically analyzed using two-way ANOVA followed by Bonferroni *post-hoc* test. ^*^: *P* ≤ 0.05 compared to transduced KASUMI-1 cells expressing empty vector. **C** Immunoblot analysis of lysates obtained from transduced KASUMI-1 cells expressing empty vector or KIT following their treatment with either vehicle (PEG) or DDP38003 (0.5 µM). Vinculin was used as a loading control. **D**-**E** Relative percent of cell count (**D**) and apoptotic cells (**E**) of KASUMI-1 following their treatment with vehicle or DDP38003 with or without: axitinib (Axi. – 25 nM), sunitinib (Sun. – 25 nM), sorafenib (Sor. – 25 nM) or lapatinib (Lap. – 5000 nM). ^a, b,c, d,e, f^: *P* ≤ 0.05 compared to KASUMI-1 cells treated with either vehicle, DDP38003, axitinib, sunitinib, sorafenib or lapatinib respectively.

To further delineate whether LSD1i-mediated downregulation of KIT contributes - at least in part - to the anti-leukemic activity of LSD1i in LSD1i-CR KIT^hi+mutant^ AML cells, we stably transduced KASUMI-1 cells with empty vector or KIT expressing vector. Indeed, exogenous expression of KIT at least partially compromised the anti-leukemic efficacy of LSD1 inhibition and was associated with heightened ERK1/2-mTOR signalling in KASUMI-1 cells (Supplementary Figure 6 A and Fig. 5A-C).

Conversely, inhibiting c-Kit (using axitinib, sunitinib or sorafenib) evidently boosted the antileukemic activity of LSD1 inhibition in LSD1i-CR KIT^hi+mutant^ AML cells (Fig. 5D-E and Supplementary Figure 6B-6 C). Nevertheless, lapatinib (an EGFR inhibitor) did not further augment the response of DDP38003 in LSD1i-CR KIT^hi+mutant^ AML cells (Fig. 5D-E). Altogether, these findings indicate that downregulation of KIT by LSD1i might - at least partially - contribute to the anti-leukemic activity of LSD1i in KIT-dependent AML cells. Furthermore, these findings emphasize the therapeutic potential of co-targeting LSD1/KIT in KIT-dependent AML cells.

### The levels of KIT mRNA positively correlate with LSD1 mRNA levels in primary AML biospecimens and LSD1 inhibition reduces KIT expression in primary biospecimens of AML patients

**Fig. 6:**
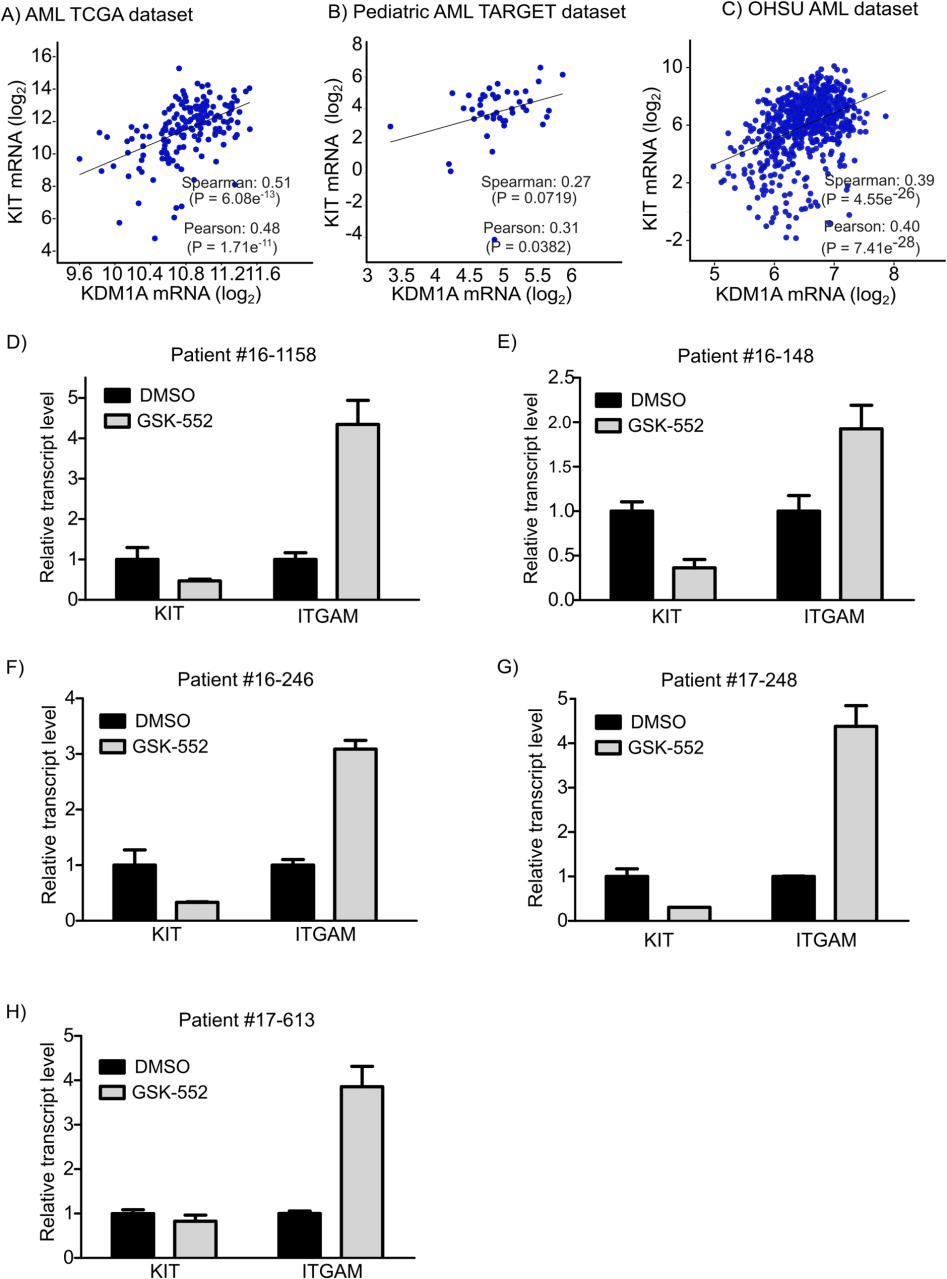
The mRNA levels of stem cell factor tyrosine kinase receptor (KIT) positively correlate with the mRNA levels of lysine specific demethylase 1 (KDM1A or LSD1) in biospecimens obtained from AML patients and LSD1 inhibition deregulates KIT transcript levels in primary human AML samples. **A** Correlation analysis of KDM1A and KIT mRNA expression (log_2_ RNA-Seq V2) in primary AML biospecimens obtained from AML TCGA dataset (cBioportal, NJEM, 2013) (*n* = 173). (Spearman: 0.51 [*P* = 6.08e^− 13^] and Pearson: 0.48 [*P* = 1.71e^− 11^]). **B** Correlation analysis of KDM1A and KIT mRNA expression (log_2_ RNA-Seq RPKM) in primary AML specimens obtained from Pediatric AML TARGET dataset (cBioportal, 2018) (*n* = 45) (Spearman: 0.27 [*P* = 0.0719] and Pearson: 0.31 [*P* = 0.0382]). **C** Correlation analysis of KDM1A and KIT mRNA expression (log2 RNA-Seq RPKM) of primary AML biospecimens obtained from OHSU Dataset (cBioportal, Cancer Cell, 2022) (*n* = 671) (Spearman: 0.39 [*P* = 4.55e^− 26^] and Pearson: 0.40 [*P* = 7.41e^− 28^]). **D**-**H**) Relative transcript levels of KIT and ITGAM assessed using RNA-Sequencing (GSE190785) in primary patient-derived AML biospecimens: Patient #16-1158 (**D**), Patient #16–148 (**E**), Patient #16–246 (**F**), Patient #17–248 (**G**) and Patient #17–613(H)) 24 h following their treatment with either DMSO or GSK2879552 (GSK-552) (500 nM)

We asked whether the transcript levels of KIT correlated with that of LSD1 in primary biospecimens obtained from AML patients. Inspecting the AML TCGA dataset (NEJM, 2013) (*n* = 173) showed that the levels of KIT mRNA significantly positively correlated with the mRNA levels of LSD1 in primary human AML biospecimens (Spearman: 0.51 [*P* = 6.08e^− 13^] and Pearson: 0.48 [*P* = 1.71e^− 11^]) (Fig. 6A). In the Pediatric AML TARGET dataset (2018) (which included data from small number of AML patients (*n* = 45)), the Spearman and Pearson correlation coefficients of LSD1 versus KIT were 0.27 [*P* = 0.0719] and 0.31 [*P* = 0.0382] respectively (Fig. 6B). Exploiting OHSU AML dataset (Cancer cell, 2022) which comprised data from 671 patients revealed that there was a significant positive correlation between KIT and LSD1 transcript levels (Spearman: 0.39 [*P* = 4.55e^− 26^] and Pearson: 0.40 [*P* = 7.41e^− 28^]) (Fig. 6C). It is worth mentioning that LSD1 mRNA levels did not correlate with the transcript levels of other tyrosine kinase receptors such as AXL and MET or c-Kit ligand (Supplementary Figure 7 A-7 F).

Next, we sought to investigate the potential effect of LSD1 inhibition on KIT expression in primary AML biospecimens *ex vivo*. To this end, we exploited publicly available RNA-seq dataset (GSE190785) of primary AML biospecimens treated with vehicle or GSK2879552 (a selective and irreversible LSD1 inhibitor) for 24 h [41]. Except for modest inhibitory effects on KIT expression (20% downregulation) in one primary patient-derived AML sample, LSD1 inhibition robustly downregulated KIT expression (by 50%-70%) and upregulated the expression ITGAM – one of the direct target genes of LSD1 – in four out of the five tested primary AML biospecimens (Fig. 6D and H). Thus, the present findings suggest that LSD1 inhibition downregulates KIT expression in primary patient-derived AML biospecimens.

## Discussion

We previously reported that mTORC1 signalling is differentially modulated in AML cells following their treatment with LSD1is [3]. mTORC1 signalling is triggered in LSD1i-PR/IR AML cells and is abrogated in LSD1i-CR AML cells [3]. We and others also found that co-targeting LSD1/mTOR elicited superior anti-leukemic activity compared to monotherapies [3, 21]. In the present study, further interrogation of the baseline transcriptomic differences between LSD1i-PR/IR AML cells and LSD1i-CR AML cells revealed that KIT is selectively overexpressed (in logarithmic scale) in LSD1i-CR AML cells compared to LSD1i-PR/IR AML cells. These results were further confirmed using RT-qPCR, immunoblot and flow cytometric analyses. It is worth noting that we validated the reproducibility of our results (which were linked to DDP38003 and shRNAs against LSD1) in other studies which also reported heterogeneous responses of AML cells to other pharmacological LSD1is as RN-1 and GSK-552 [17, 18]. Indeed, KIT was significantly overexpressed in AML cells which are sensitive to RN-1 and GSK-552. KIT proto-oncogene receptor tyrosine kinase plays fundamental roles in maintaining the survival, proliferation and stemness of cancer cells [42–45]. Coherently, cancer cells with pluripotent stemness properties were found to be selectively susceptible to LSD1i [46]. Nonetheless, further studies are warranted to inspect whether KIT overexpression is also evident in other types of cancer (other than AMLs) which are responsive to LSD1i.

Interestingly, pharmacological LSD1is as well as genetic LSD1 knock down repressed KIT transcription in LSD1i-PR/IR AML cells and LSD1i-CR AML cells. In harmony with our data, Fiskus and colleagues also reported that LSD1 KO via CRISPR-Cas9 reduced the percent of c-Kit^+^ expressing AML and post-myeloproliferative neoplasm secondary AML cells [47]. Inspecting ENCODE and FANTOM5 predicted LSD1 to be one of the transcription factors binding to KIT enhancer. LSD1 ChIP-Seq and ChIP-qPCR analyses confirmed the binding of LSD1 to regions in the vicinity of the TSS of KIT gene. LSD1i increased the occupancy of the repressive H3K9me2 marks close to the TSS of KIT gene. Our findings are in line with that of Metzger and colleagues who reported that LSD1 demethylated H3K9me2 histone marks to favour the transcription of androgen-receptor [2]. Estrogen-related receptor α (ERRα) also instructed LSD1 to erase repressive H3K9me2 marks at the promoters of genes involved in the migration and invasion of cancer cells [35]. LSD1-induced upregulation of tetraspanin 8 (TSPAN8) in colorectal cancer cells was associated with decreased H3K9me2 marks on the TSPAN8 promoter [48]. Maiques-Diaz and colleagues observed that rapid transcriptional changes occur post-LSD1i therapy without corresponding accumulation of H3K4me1/2 at LSD1-bound promoters and active enhancers [9]. Notably, LSD1 K661A demethylase-defective mutant and wild type LSD1 efficiently rescued LSD1 knockdown AML cells. Nonetheless, the scaffolding role of LSD1/RCOR1 and their interaction with the SNAG-domain transcription repressor GFI1 regulated the differentiation of AML cells [9]. Indeed, future systematic studies are warranted to investigate the potential role of the scaffolding versus catalytic (demethylase) function of LSD1 in regulating the transcription of KIT in AML (e.g. rescuing experiments with LSD1 demethylase dead mutant).

Although we previously reported that short term (3-day) treatment of primary human CD34^+^ cord blood cells with LSD1i did not dramatically affect their proliferation and mTOR signalling, we did not investigate the effect of LSD1i on KIT expression [3]. Nonetheless, Kerenyi and colleagues demonstrated that genetic ablation of LSD1 in hematopoietic stem cells provoked pancytopenia [49]. Indeed, LSD1^*fl/fl*^ VavCre mice had significantly lower numbers of lineage-negative Sca-1^+^ c-Kit^+^ cells and lineage-negative Sca-1^−^ c-Kit^+^ cells while sparing Sca-1^+^ c-Kit^−^ cells in the lineage-negative compartment [49].

Mutant KIT expressing LSD1i-CR AMLs - but not wild type KIT expressing LSD1i-PR/IR KIT^low^ and LSD1i-CR KIT^hi^ AMLs - were vulnerable to the genetic depletion of KIT as well as pharmacological TKIs (which target c-Kit as well). Similar findings were previously reported using imatinib mesylate (another TKI which also targets c-Kit) [50]. Indeed, LSD1i-CR KIT^hi+mutant^ AML cells such as KASUMI-1 and SKNO-1 have KIT N822K mutation which promotes constitutive activation of c-Kit signalling [48]. GDM-1 cell line harbours homozygous R420Q inactivating mutation in Casitas B-lineage lymphoma (CBL) [51]. CBL encodes for an E3 ubiquitin ligase which binds to and tags receptor tyrosine kinases (RTKs) such as c-Kit to be subsequently targeted for lysosomal degradation. Thus, this could explain the increased KIT expression in GDM-1 cell line [52]. In line with our findings, GDM-1 was highly susceptible to the anticancer activity of dasatinib (dual inhibitor of SRC family kinases (SFK) and RTK) but showed modest or minimal responses to sunitinib (RTK inhibitor), imatinib (ABL, KIT inhibitor), or PP2 (SFK inhibitor) [51].

In alignment with c-Kit acting upstream of ERK/mTOR signalling, we found that LSD1i-mediated downregulation of KIT was associated with inhibited ERK/mTOR signalling in LSD1i-CR KIT-dependent AML cells. Conversely, forced KIT overexpression activated ERK/mTOR signalling and at least partially compromised the antileukemic activity of LSD1is in LSD1i-CR KIT-dependent AML cells. Consistently, ectopic KIT expression promoted cancer resistance to 5-fluorouracil whereas induced miR-34a expression -which represses KIT transcription- sensitized cancer cells to 5-fluorouracil [53]. Intriguingly, co-targeting LSD1/c-Kit elicited heightened antileukemic activity in LSD1i-CR KIT-dependent AML cells. In the present study, we did not systematically dissect the nature of this interaction (i.e. whether additive or synergistic effect) between LSD1 inhibitor (using DDP38003) and c-Kit inhibitors (as sorafenib, sunitinib and axitinib) in KIT-mutated AMLs. Nonetheless, Curtiss and colleagues reported synergistic anti-leukemic activity of two LSD1 inhibitors: ORY-1001 or GSK-LSD1 when combined with avapritinib (c-Kit and PDGFRA inhibitor) in KIT mutated AML cell lines and primary patient samples. Delving deeper, this comboregimen synergistically blunted Myc activity which was associated with repression of cell cycle genes [33]. Yashar and colleagues reported synergistic interaction between GSK2879552 (LSD1 inhibitor) and quizartinib (FLT3 and c-Kit inhibitor) in primary AML patients regardless of FLT3 mutation status [41, 54]. This combination disrupted the binding of STAT5, LSD1, and GFI1 binding at the MYC blood super-enhancer and increased repressive H3K9me1 methylation at MYC target genes [41]. Noting limited availability of PDX models of KIT mutant AMLs, Curtiss et al. evaluated the tolerability of two-week treatment with ORY-1001 (LSD1 inhibitor) and avapritinib (c-Kit and PDGFRA inhibitor) in healthy mice. Intriguingly, the tested comboregimen did not affect the body weight, WBCs and hemoglobin of the treated mice [33]. Nonetheless, in vivo preclinical testing of the tolerability and efficacy of LSD1i + c-Kit inhibitor combination therapy is warranted in PDX models of KIT-mutated AMLs.

Finally, we validated that KIT mRNA levels positively correlated with that of LSD1 in primary biospecimens obtained from AML patients in at least two independent AML datasets (AML TCGA and BeatAML.2 datasets). Furthermore, we demonstrated that LSD1 inhibition repressed KIT transcription in primary patient-derived AML biospecimens ex vivo.

## Conclusions

In conclusion, the present study emphasizes that KIT-dependent AML patients are most likely to benefit from LSD1i therapy and underscores the therapeutic potential of co-targeting LSD1/KIT in this subpopulation of AML patients. Assessing baseline KIT levels might serve as a potential biomarker to guide clinicians towards more objective patient selection for LSD1i therapy. To the best of our knowledge, this study is the first which demonstrated that LSD1 epigenetically modulated KIT transcription and this was associated with relieved occupancy of repressive H3K9me2 histone marks in proximity to the TSS of KIT.

## Supplementary Information

Below is the link to the electronic supplementary material.

Supplementary Material 1

## Data Availability

RNA-seq data of AML cell lines treated with either vehicle or GSK-552 were obtained from the NCBI gene expression omnibus (GEO) with the accession GSE125112. We utilized publicly deposited data resources produced by the Cancer Cell Line Encyclopedia (CCLE) through the Cancer Dependency Map (DepMap, 21Q2) portal ( [https://depmap.org/portal/](https:/depmap.org/portal) ),  Genomics of Drug Sensitivity in Cancer database ( [https://www.cancerrxgene.org/](https:/www.cancerrxgene.org) )  and cistrome ( [http://cistrome.org/db/#/](http:/cistrome.org/db) ).

## Abbreviations

AML: Acute myeloid leukemia
ERK: Extracellular signal regulated kinase
KIT: Stem cell factor tyrosine kinase receptor
KDM1A/LSD1: Lysine specific demethylase-1
mTOR: Mammalian target of rapamycin
