# Supplementary material for: KIT-dependent acute myeloid leukemias are responsive to LSD1 inhibition
Source: Clin Epigenetics. 2026 May 14;18:80. doi: 10.1186/s13148-026-02098-w (PMC13173958; doi:10.1186/s13148-026-02098-w)

Supplemental Tables

Table.S1. List of short hairpins used in this study

Target of shRNAs	Oligos sequence/ Catalogue number/ Source
Scramble shRNAs	AGTACGCGAAGAATACTATCGA
shRNAs against LSD1 #1	AAGTGATACTGTGCTTGTCCAC
shRNAs against LSD1 #2	ATCTCAGAAGATGAGTATTATT
TRIPZ Inducible Lentiviral shCtrl	Gift from Alessandro Verrecchia
TRIPZ Inducible Lentiviral shRNA#1 for KIT	RHS4696-200685599
TRIPZ Inducible Lentiviral shRNA#2 for KIT	RHS4696-200698892

Table.S2. List of RT-qPCR primers used in this study

Primer	Primers' sequence
LSD1	F:AGACGACAGTTCTGGAGGGTA
	R: TCTTGAGAAGTCATCCGGTCA
CD117	F: CGTGGAAAAGAGAAAACAGTCA
	R: CACCGTGATGCCAGCTATTA
GAPDH	F: TTCGCTCTCTGCTCCTCCTG
	R: CCTAGCCTCCCGGGTTTCTC

Table.S3. List of ChIP-qPCR primers used in this study

Primer	Primers' sequence	Distance from KIT TSS
#1	F: TGGTGAGTGCAGGAATCCAA<br>R: ACCAACACAAGGTAGGCCG	4.261 Kb
#2	F:TGCATGTTTATCTGCTTGGCTC<br>R: GCAGACATTCCCCCAACCTAA	5.735 Kb

Table.S4. List of top 50 differentially expressed genes in KASUMI-1 versus OCI-AML3 AML cells (Cut-off Log2FC = | 1 | and adjusted P value ≤ 0.05 (GSE125112).

Gene symbol	log_2FC	P_{adj}	Description
EPCAM	16.30071	3.96E-43	epithelial cell adhesion molecule [Source:HGNC Symbol;Acc:HGNC:11529]
PRSS21	15.99248	1.50E-41	protease, serine, 21 (testisin) [Source:HGNC Symbol;Acc:HGNC:9485]
TES	15.92682	3.09E-41	testin LIM domain protein

			[Source:HGNC Symbol;Acc:HGNC:14620]
COL4A5	15.75584	2.44E-40	collagen, type IV, alpha 5 [Source:HGNC Symbol;Acc:HGNC:2207]
KIT	15.75298	1.83E-32	v-kit Hardy-Zuckerman 4 feline sarcoma viral oncogene homolog [Source:HGNC Symbol;Acc:HGNC:6342]
MYO18B	15.68159	6.41E-40	myosin XVIIIB [Source:HGNC Symbol;Acc:HGNC:18150]
SLCO5A1	15.45617	1.28E-38	solute carrier organic anion transporter family, member 5A1 [Source:HGNC Symbol;Acc:HGNC:19046]
WIPF3	15.40786	3.35E-38	WAS/WASL interacting protein family, member 3 [Source:HGNC Symbol;Acc:HGNC:22004]
ITM2A	15.19343	2.16E-37	integral membrane protein 2A [Source:HGNC Symbol;Acc:HGNC:6173]
FBN2	15.1453	8.11E-48	fibrillin 2 [Source:HGNC Symbol;Acc:HGNC:3604]
TRPM6	15.10971	6.22E-37	transient receptor potential cation channel, subfamily M, member 6 [Source:HGNC Symbol;Acc:HGNC:17995]
EVC	15.006	1.93E-36	Ellis van Creveld protein [Source:HGNC Symbol;Acc:HGNC:3497]
DSG2	14.96295	3.37E-36	desmoglein 2 [Source:HGNC Symbol;Acc:HGNC:3049]
ZNF711	14.77574	3.07E-35	zinc finger protein 711 [Source:HGNC Symbol;Acc:HGNC:13128]
CD96	14.69524	4.54E-35	CD96 molecule [Source:HGNC Symbol;Acc:HGNC:16892]
TCAM1P	14.53544	3.77E-34	testicular cell adhesion molecule 1, pseudogene [Source:HGNC Symbol;Acc:HGNC:30707]
EVC2	14.48875	6.64E-34	Ellis van Creveld syndrome 2 [Source:HGNC Symbol;Acc:HGNC:19747]
ZNF160	14.42182	1.96E-33	zinc finger protein 160 [Source:HGNC Symbol;Acc:HGNC:12948]
RANBP17	14.41947	1.46E-33	RAN binding protein 17 [Source:HGNC Symbol;Acc:HGNC:14428]
VSTM1	14.34289	4.50E-33	V-set and transmembrane domain

			containing 1 [Source:HGNC Symbol;Acc:HGNC:29455]
SPINT2	14.14142	6.84E-42	serine peptidase inhibitor, Kunitz type, 2 [Source:HGNC Symbol;Acc:HGNC:11247]
APP	14.1077	2.87E-82	amyloid beta (A4) precursor protein [Source:HGNC Symbol;Acc:HGNC:620]
GALNT14	14.04705	9.15E-32	polypeptide N-acetylgalactosaminyltransferase 14 [Source:HGNC Symbol;Acc:HGNC:22946]
ENPP4	13.89987	4.81E-31	ectonucleotide pyrophosphatase/phosphodiesterase 4 (putative) [Source:HGNC Symbol;Acc:HGNC:3359]
MCTP2	13.54672	1.39E-29	multiple C2 domains, transmembrane 2 [Source:HGNC Symbol;Acc:HGNC:25636]
SLC5A5	13.53586	2.39E-29	solute carrier family 5 (sodium/iodide cotransporter), member 5 [Source:HGNC Symbol;Acc:HGNC:11040]
CTC-546K23.1	13.51185	3.14E-29	NA
ARRDC4	13.42873	2.57E-29	arrestin domain containing 4 [Source:HGNC Symbol;Acc:HGNC:28087]
OPRK1	13.3881	8.17E-29	opioid receptor, kappa 1 [Source:HGNC Symbol;Acc:HGNC:8154]
MIR4458HG	13.38515	8.73E-29	MIR4458 host gene [Source:HGNC Symbol;Acc:HGNC:49008]
STK32B	13.26016	2.93E-28	serine/threonine kinase 32B [Source:HGNC Symbol;Acc:HGNC:14217]
GSTM1	13.17429	7.73E-28	glutathione S-transferase mu 1 [Source:HGNC Symbol;Acc:HGNC:4632]
CYTL1	13.15312	8.18E-28	cytokine-like 1 [Source:HGNC Symbol;Acc:HGNC:24435]
B3GALNT1	13.1344	1.05E-27	beta-1,3-N-acetylgalactosaminyltransferase 1 (globoside blood group) [Source:HGNC Symbol;Acc:HGNC:918]
PAWR	13.11958	2.82E-36	PRKC, apoptosis, WT1, regulator [Source:HGNC Symbol;Acc:HGNC:8614]
CCSER1	13.0459	2.76E-27	coiled-coil serine-rich protein 1

			[Source:HGNC Symbol;Acc:HGNC:29349]
HIST1H3G	12.99669	8.00E-37	histone cluster 1, H3g [Source:HGNC Symbol;Acc:HGNC:4772]
LINC00689	12.95711	5.97E-27	long intergenic non-protein coding RNA 689 [Source:HGNC Symbol;Acc:HGNC:27217]
ZNF329	12.95304	5.54E-27	zinc finger protein 329 [Source:HGNC Symbol;Acc:HGNC:14209]
TMIGD2	12.94884	2.19E-35	transmembrane and immunoglobulin domain containing 2 [Source:HGNC Symbol;Acc:HGNC:28324]
ZNF606	12.93497	8.40E-27	zinc finger protein 606 [Source:HGNC Symbol;Acc:HGNC:25879]
ZSCAN18	12.86431	1.77E-26	zinc finger and SCAN domain containing 18 [Source:HGNC Symbol;Acc:HGNC:21037]
HOXD13	12.85266	1.70E-26	homeobox D13 [Source:HGNC Symbol;Acc:HGNC:5136]
SLC28A3	12.82207	2.24E-26	solute carrier family 28 (concentrative nucleoside transporter), member 3 [Source:HGNC Symbol;Acc:HGNC:16484]
MPO	12.81833	0	myeloperoxidase [Source:HGNC Symbol;Acc:HGNC:7218]
RP11-251M1.1	12.78544	3.70E-26	NA
DEPDC7	12.75824	4.66E-26	DEP domain containing 7 [Source:HGNC Symbol;Acc:HGNC:29899]
RP11-1134I14.2	12.73847	7.80E-26	NA
CLEC5A	12.7149	1.78E-83	C-type lectin domain family 5, member A [Source:HGNC Symbol;Acc:HGNC:2054]

Legends of Supplementary Figures

Supplementary Figure.1. Human acute myeloid leukemia (AML) cells differentially respond to DDP38003, a selective and potent irreversible LSD1 inhibitor.

A-F) Relative percent of cell count of KASUMI-1 (A), GDM-1 (B), HEL (C), ML-2 (D), MOLM-13 (E) and HL60 (F) cells 7 days following their treatment with either vehicle or DDP38003 (0.5 µM). *: P≤0.05 compared to vehicle treated cells assessed using Student's t test.

G-H) Representative flowcytometry histogram (G) of cell cycle distribution in M-07e cells 7 days following their treatment with either vehicle or DDP38003 (0.5 µM).

H) Percent of the proportion/distribution of M-07e cells in different phases of the cell cycle (G_0/G_1, S and G_2/M) 7 days following their treatment with either vehicle or DDP38003 (0.5 µM).

I-J) Representative flowcytometry histogram (I) of cell cycle distribution in OCI-AML5 cells 7 days following their treatment with either vehicle or DDP38003 (0.5 µM). J) Percent of the proportion/distribution of OCI-AML5 cells in different phases of the cell cycle (G_0/G_1, S and G_2/M) 7 days following their treatment with either vehicle or DDP38003 (0.5 µM).

Supplementary Figure.2. The mRNA and copy number of stem cell factor tyrosine kinase receptor (KIT) and the expression pattern of other tyrosine kinase receptors in human acute myeloid leukemia (AML) cell lines which are sensitive (CR) or partially responsive (PR) to selective irreversible LSD1 inhibitors.

A) Relative KIT mRNA level in AML cells assessed using RT-qPCR.

B-F) Box plot depicting the CCLE KIT copy number (B), platelet derived growth factor receptor beta (PDGFRβ) (C), AXL tyrosine kinase receptor (C), epidermal growth factor tyrosine kinase receptor (EGFR) (D) and MET tyrosine kinase receptor in RN-1 CR and RN-1 PR AML cell lines. ns: non-significant.

Supplementary Figure.3. Stem cell factor tyrosine kinase receptor (KIT) is significantly overexpressed in GSK2879552 (GSK-552, a selective irreversible LSD1 inhibitor) in completely responsive (CR) AML cell lines compared to partially responsive (PR) AML cells.

A-E) Box plot depicting the expression levels of stem cell factor tyrosine kinase receptor (KIT) $\log_2$ (TPM+1) (A), c-Kit reverse protein phase array (RPPA) ($\log_2$) (B), AXL tyrosine kinase receptor (C), epidermal growth factor tyrosine kinase receptor (EGFR) (D) and FLT3 receptor (E) in GSK-552 CR and GSK-552 PR AML cell lines. *: P≤0.05 compared to GSK-552 PR AML cells. ns: non-significant.

Supplementary Figure. 4. The antileukemic activity of LSD1 inhibition is associated with increased apoptosis, macrophage-lineage differentiation and reduced KIT transcription.

A) Relative KIT mRNA level in KASUMI-1 cells transduced with shControl and shLSD1 assessed using RT-qPCR. Data were statistically analyzed using Student's t test, *: P≤0.05 compared to shCtrl transduced KASUMI-1 cells.

B) Percent of cell count of murine c-Kit$^+$ MLL-AF9 AML cells 144h following their treatment with either vehicle or DDP38003 (0.5 µM). *: P≤0.05 compared to vehicle treated cells assessed using One way ANOVA followed by Bonferonni *post hoc* test.

C) Representative phase contrast images of murine c-Kit$^+$ MLL-AF9 AML cells 144h following their treatment with either vehicle or DDP38003 (0.1 and 0.5 μM).
D-E) Flowcytometry dot plots (D) and quantitation (E) showing the effect of 6 days of DDP38003 treatment on the viability/apoptosis of murine c-Kit$^+$ MLL-AF9 AML cells assessed using Annexin V/PI staining.
F) Representative phase contrast images of cytospin preparations recovered after 3 and 6 days of treatment with murine c-Kit$^+$ MLL-AF9 AML cells treated with either vehicle and DDP38003 (0.5 μM) and stained with May Grunwald Giemsa stain.
G) Normalized KIT mRNA levels of murine c-Kit$^+$ MLL-AF9 AML cells treated with the indicated concentrations of DDP38003 for 6 days assessed using RT-qPCR. Data were statistically analyzed using one way ANOVA followed by Bonferonni post-hoc test. *: P≤0.05 compared to vehicle-treated cells.
H-J) Relative normalized RPPA levels of c-Kit (H), MET (I), and AXL (J) tyrosine kinase receptors in KASUMI-1 cells following LSD1 inhibition using ORY-1001 (12 nM).

Supplementary Figure 5. LSD1 (KDM1A) binds close to KIT TSS and modulates H3K27 acetylation in acute myeloid leukemia (AML) cells.
A) Cistrome LSD1 ChIP-Seq tracks depicting KIT transcription start site (TSS) in SKNO-1 cells (McGrath et al. 2016 dataset).
B-C) LSD1 ChIP-qPCR analysis of THP-1 (B) and KASUMI-1 (C) AML cells. Data were statistically analyzed using Student’s t test, *: P≤0.05 compared to IgG immunoprecipitated AML cells.
D-E) H3K27Ac ChIP-qPCR analysis of THP-1 (D) and SKNO-1 (E) cells treated with either vehicle (PEG) or DDP38003 (0.5 μM). Data were statistically analyzed using Student’s t test, *: P≤0.05 compared to vehicle-treated cells.

Supplementary Figure 6. Co-targeting LSD1 and c-Kit elicit superior anticancer activity in LSD1i-CR KIT dependent AML cells.
A)Relative levels of c-Kit protein assessed using FACS in KASUMI-1 cells transduced with empty vector (EV) and vector overexpressing c-Kit (KIT OE). *: P≤0.05 compared to EV transduced KASUMI-1 cells assessed using Student’s t test.
B-C) Percent of cellular ATP levels assessed using Cell Titer-Glo™ Luminescent Cell Viability assay of KASUMI-1 cells following their treatment with the indicated concentrations of vehicle or DDP38003 with or without the indicated concentrations of sorafenib (B) or sunitinib (C). *: P≤0.05 compared to vehicle treated cells assessed using two-way ANOVA followed by Bonferroni’s *post hoc* test.

Supplementary Figure 7. Co-expression analysis of the transcript levels of lysine specific demethylase 1 (KDM1A or LSD1) and other tyrosine kinase receptors or c-Kit ligand using cBioportal in primary biospecimens obtained from AML patients.
A-C) Correlation analysis of the mRNA expression ($\log_2$ RNA-Seq RPKM) of KDM1A versus AXL (A), KDM1A versus MET (B) and KDM1A versus cKit ligand (KITLG) (C) in primary AML biospecimens obtained from Pediatric AML TARGET dataset

(cBioportal, 2018) (n = 45). Spearman’s and Pearson’s correlation coefficients and the corresponding P values are indicated.

D-F) Correlation analysis of the mRNA expression (log2 RNA-Seq RPKM) of KDM1A versus AXL (D), KDM1A versus MET (E) and KDM1A versus cKit ligand (KITLG) (F) in primary AML biospecimens obtained from OHSU Dataset (cBioportal, Cancer Cell, 2022) (n = 671). Spearman’s and Pearson’s correlation coefficients and the corresponding P values are indicated.

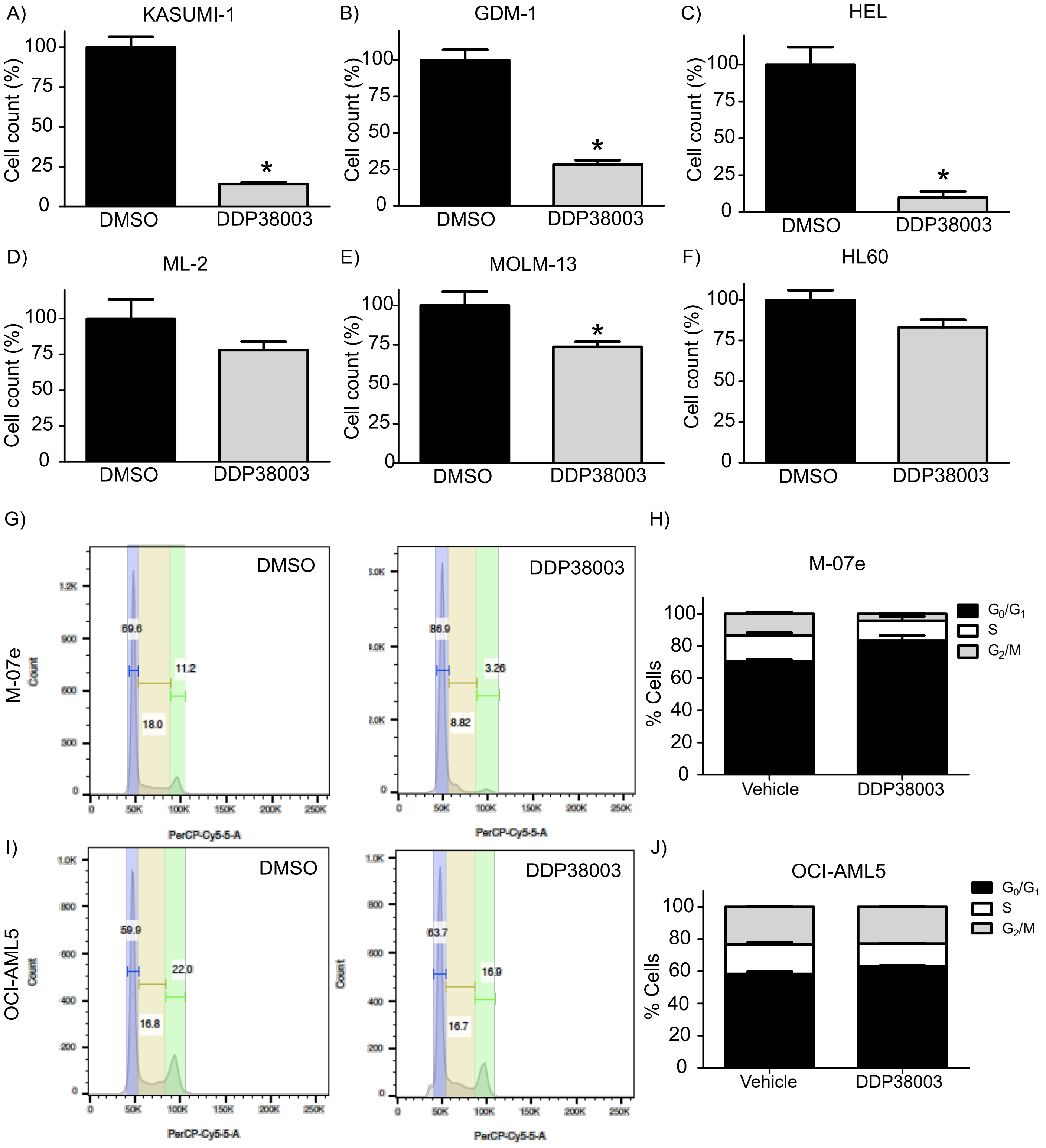
A)
KASUMI-1
Cell count (%)
100
75
50
25
0
DMSO
DDP38003
*
B)
GDM-1
C)
HEL
D)
ML-2
E)
MOLM-13
F)
HL60
G)
M-07e
DMSO
DDP38003
Count
PerCP-Cy5-5-A
69.6
18.0
11.2
86.9
8.82
3.26
H)
M-07e
% Cells
Vehicle
G0/G1
S
G2/M
I)
OCI-AML5
59.9
16.8
22.0
63.7
16.7
16.9
J)
OCI-AML5

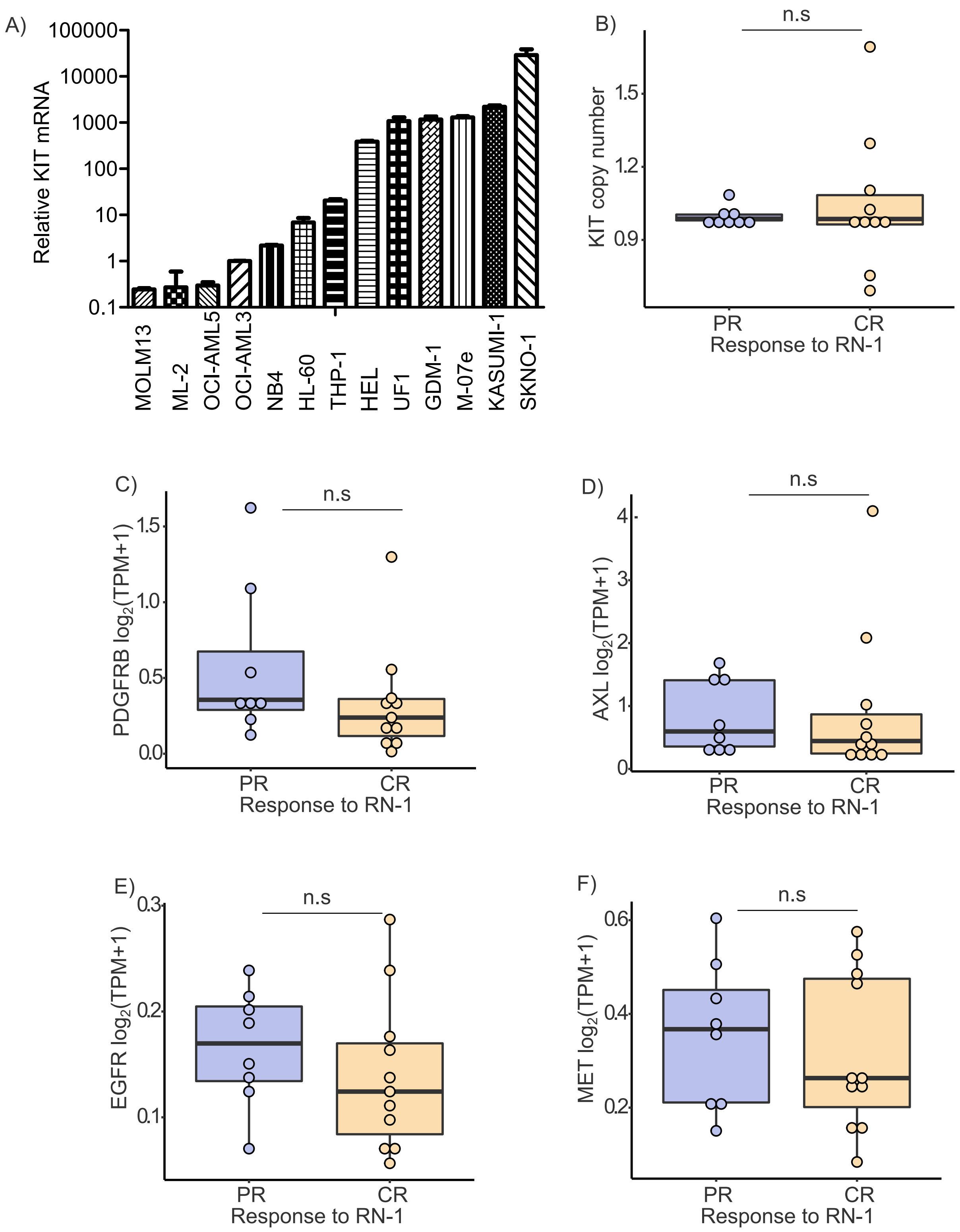
A)
Relative KIT mRNA
100000
10000
1000
100
10
1
0.1
MOLM13
ML-2
OCI-AML5
OCI-AML3
NB4
HL-60
THP-1
HEL
UF1
GDM-1
M-07e
KASUMI-1
SKNO-1
B)
n.s
KIT copy number
1.5
1.2
0.9
PR
CR
Response to RN-1
C)
n.s
PDGFRB log2(TPM+1)
1.5
1.0
0.5
0.0
PR
CR
Response to RN-1
D)
n.s
AXL log2(TPM+1)
4
3
2
1
0
PR
CR
Response to RN-1
E)
n.s
EGFR log2(TPM+1)
0.3
0.2
0.1
PR
CR
Response to RN-1
F)
n.s
MET log2(TPM+1)
0.6
0.4
0.2
PR
CR
Response to RN-1

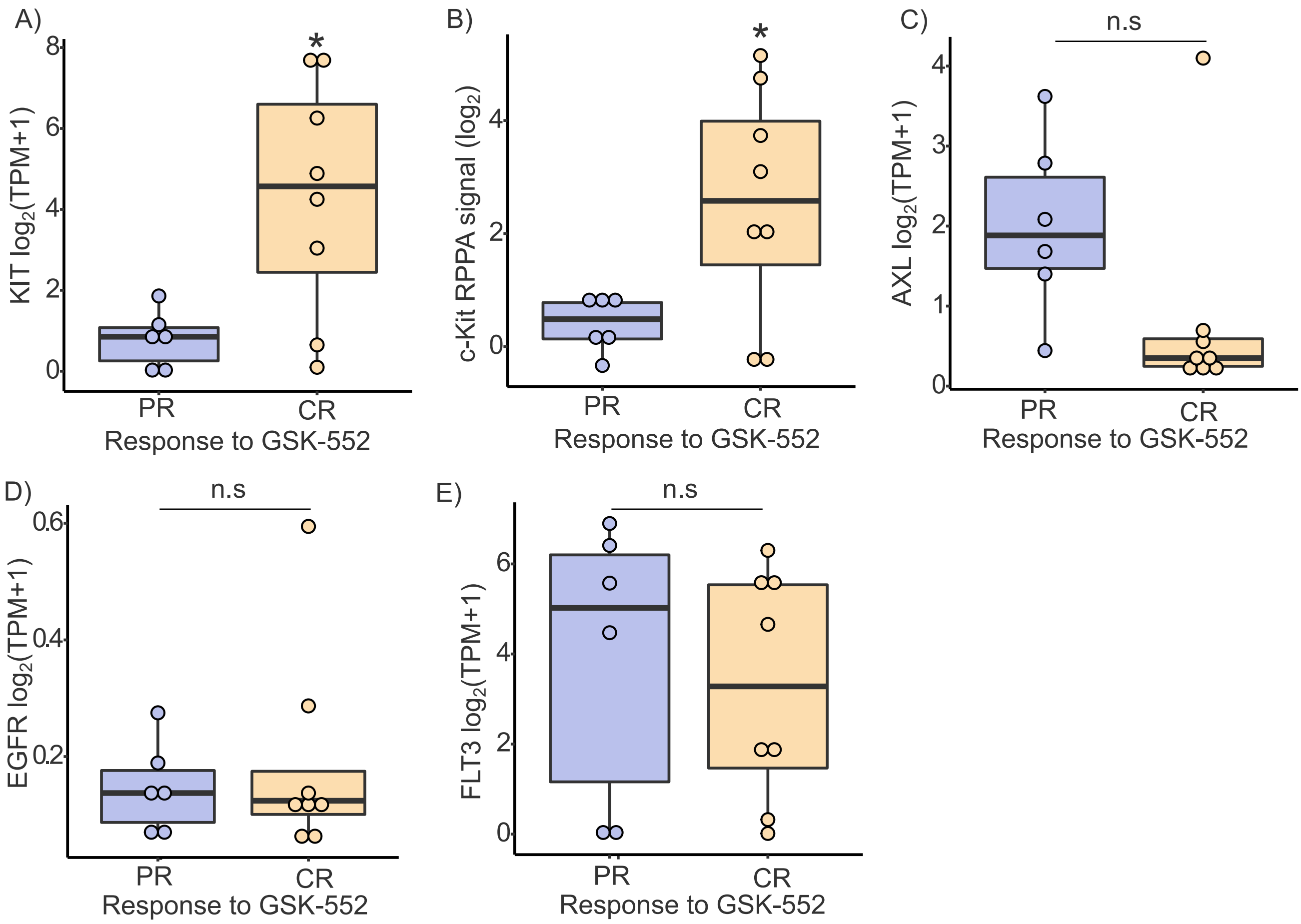

A)
KIT log_2(TPM+1)
0
2
4
6
8
*
PR
CR
Response to GSK-552
B)
c-Kit RPPA signal (log_2)
0
2
4
*
PR
CR
Response to GSK-552
C)
n.s
AXL log_2(TPM+1)
0
1
2
3
4
PR
CR
Response to GSK-552
D)
n.s
EGFR log_2(TPM+1)
0.2
0.4
0.6
PR
CR
Response to GSK-552
E)
n.s
FLT3 log_2(TPM+1)
0
2
4
6
PR
CR
Response to GSK-552

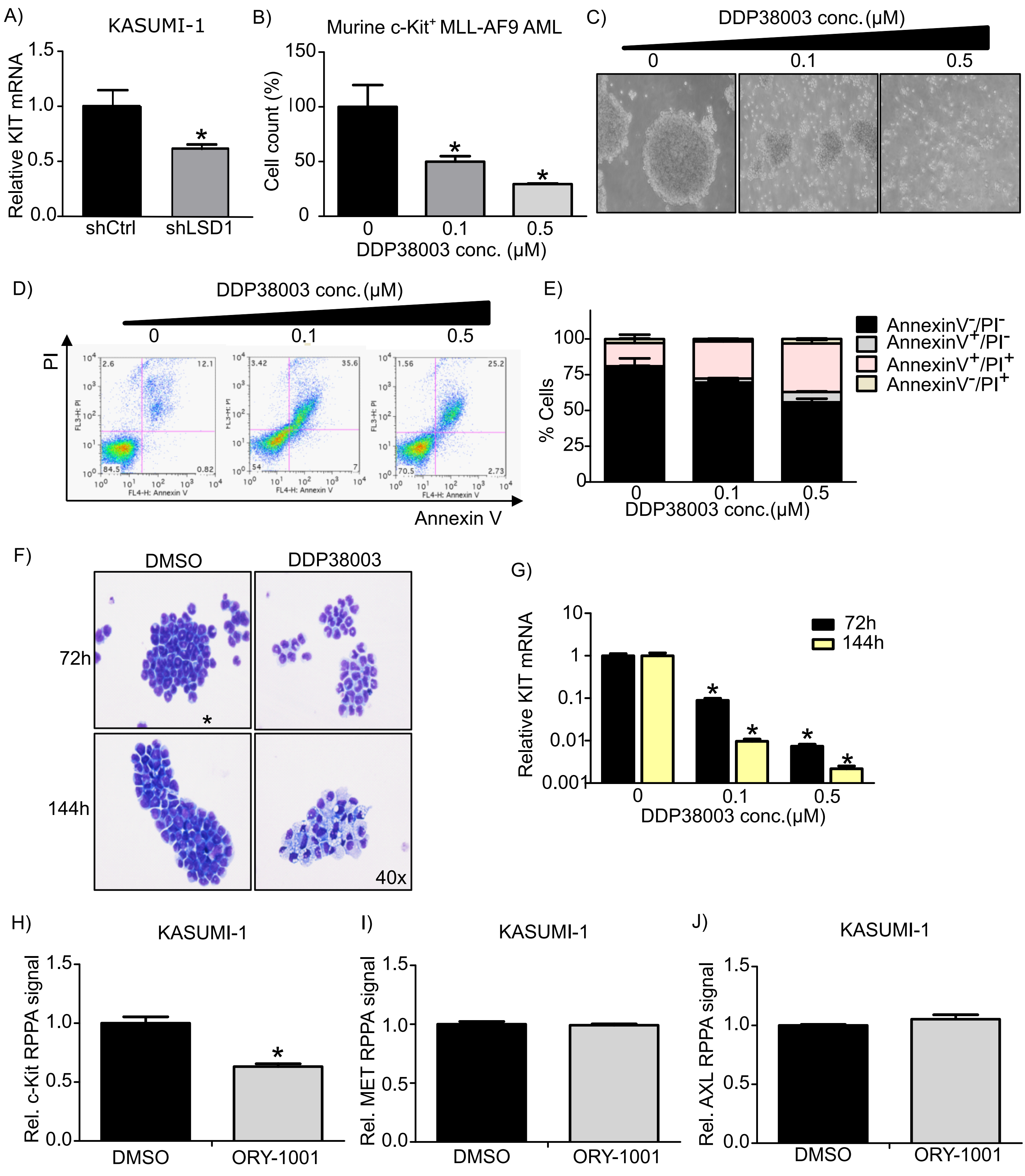
A)
KASUMI-1
Relative KIT mRNA
1.5
1.0
0.5
0.0
shCtrl
shLSD1
B)
Murine c-Kit+ MLL-AF9 AML
Cell count (%)
150
100
50
0
0
0.1
0.5
DDP38003 conc. (μM)
C)
DDP38003 conc. (μM)
0
0.1
0.5
D)
DDP38003 conc. (μM)
0
0.1
0.5
PI
Annexin V
E)
% Cells
100
75
50
25
0
0
0.1
0.5
DDP38003 conc.(μM)
AnnexinV-/PI-
AnnexinV+/PI-
AnnexinV+/PI+
AnnexinV-/PI+
F)
DMSO
DDP38003
72h
144h
40x
G)
Relative KIT mRNA
10
1
0.1
0.01
0.001
72h
144h
0
0.1
0.5
DDP38003 conc.(μM)
H)
KASUMI-1
Rel. c-Kit RPPA signal
1.5
1.0
0.5
0.0
DMSO
ORY-1001
I)
KASUMI-1
Rel. MET RPPA signal
1.5
1.0
0.5
0.0
DMSO
ORY-1001
J)
KASUMI-1
Rel. AXL RPPA signal
1.5
1.0
0.5
0.0
DMSO
ORY-1001

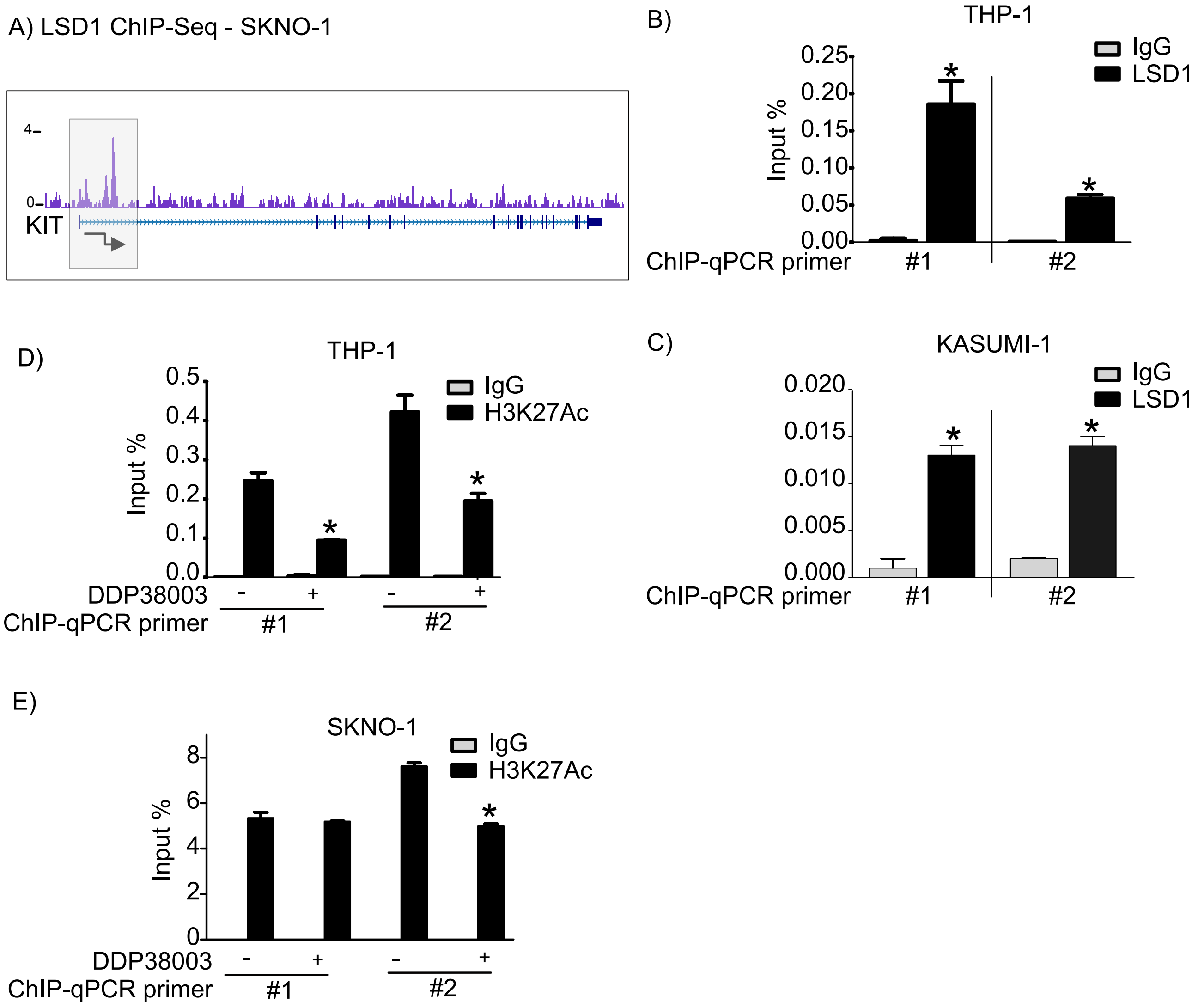
A) LSD1 ChIP-Seq - SKNO-1
4
0
KIT
B)
THP-1
IgG
LSD1
Input %
0.25
0.20
0.15
0.10
0.05
0.00
*
*
ChIP-qPCR primer
#1
#2
D)
THP-1
IgG
H3K27Ac
Input %
0.5
0.4
0.3
0.2
0.1
0.0
*
*
DDP38003
-
+
-
+
ChIP-qPCR primer
#1
#2
C)
KASUMI-1
IgG
LSD1
0.020
0.015
0.010
0.005
0.000
*
*
ChIP-qPCR primer
#1
#2
E)
SKNO-1
IgG
H3K27Ac
Input %
8
6
4
2
0
*
DDP38003
-
+
-
+
ChIP-qPCR primer
#1
#2

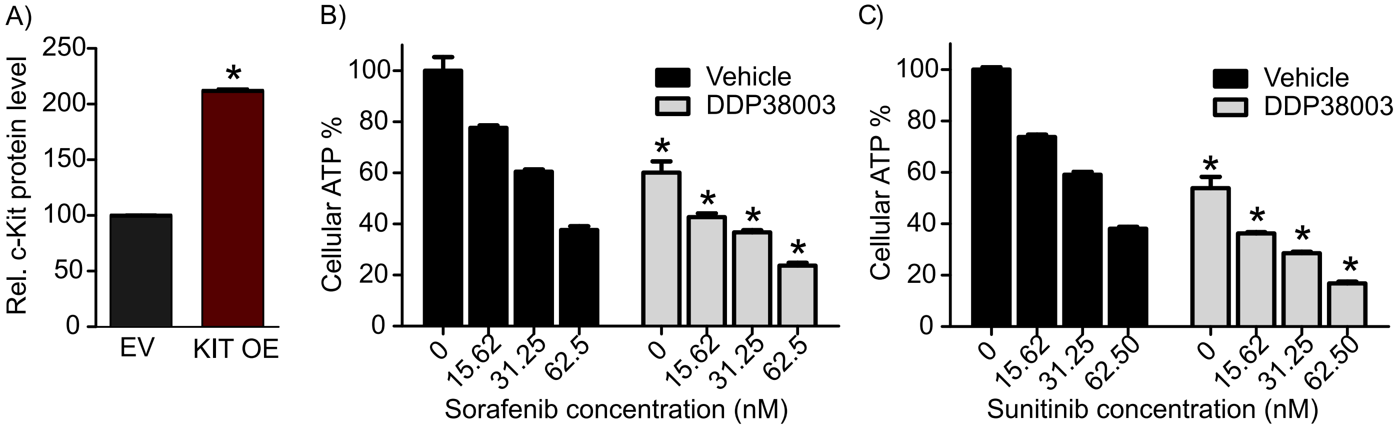
A)
Rel. c-Kit protein level
250
200
150
100
50
0
*
EV
KIT OE
B)
Cellular ATP %
100
80
60
40
20
0
Vehicle
DDP38003
*
*
*
*
0
15.62
31.25
62.5
0
15.62
31.25
62.5
Sorafenib concentration (nM)
C)
Cellular ATP %
100
80
60
40
20
0
Vehicle
DDP38003
*
*
*
*
0
15.62
31.25
62.50
0
15.62
31.25
62.50
Sunitinib concentration (nM)

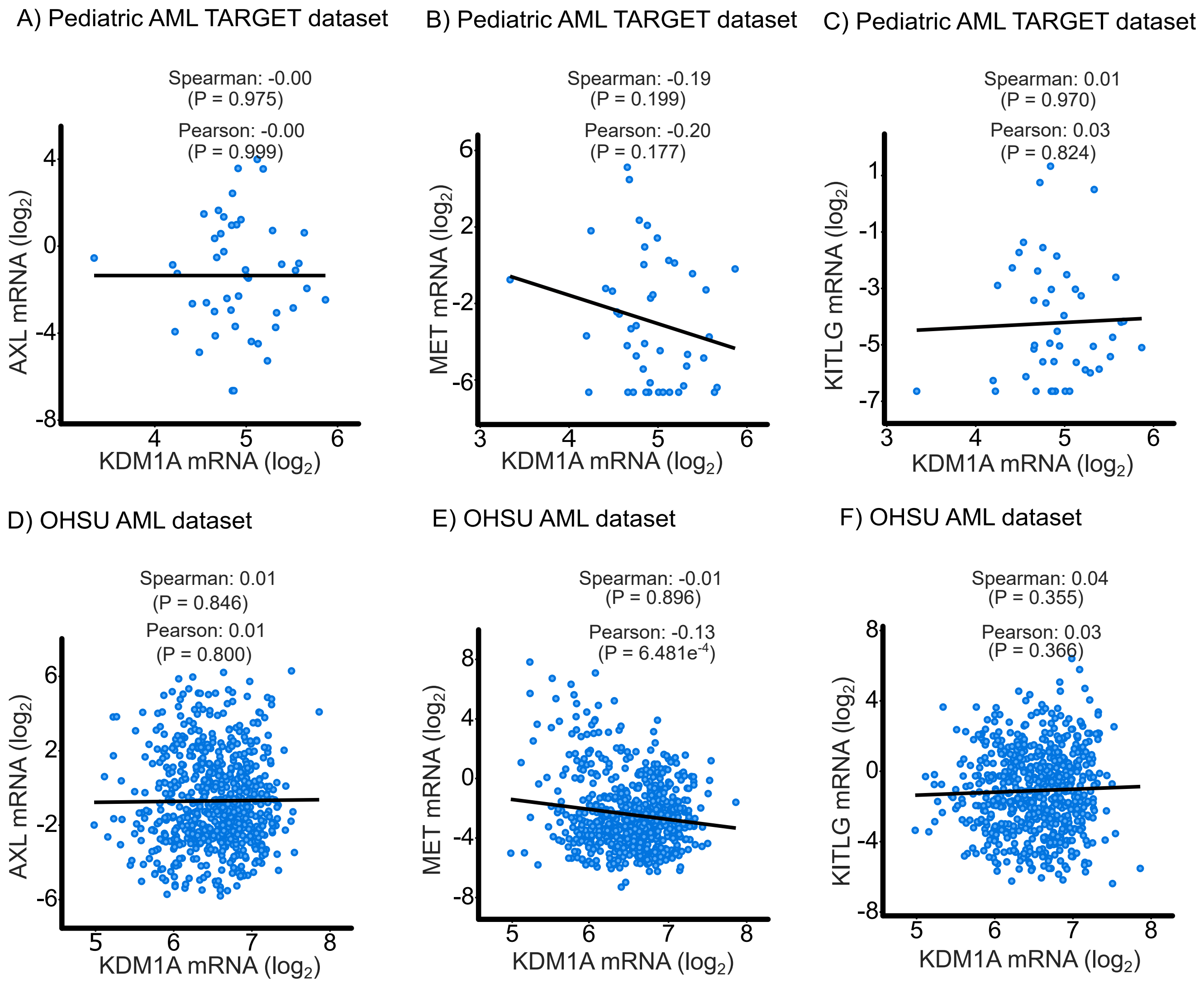

A) Pediatric AML TARGET dataset
Spearman: -0.00
(P = 0.975)
Pearson: -0.00
(P = 0.999)
AXL mRNA (log_2)
KDM1A mRNA (log_2)
B) Pediatric AML TARGET dataset
Spearman: -0.19
(P = 0.199)
Pearson: -0.20
(P = 0.177)
MET mRNA (log_2)
KDM1A mRNA (log_2)
C) Pediatric AML TARGET dataset
Spearman: 0.01
(P = 0.970)
Pearson: 0.03
(P = 0.824)
KITLG mRNA (log_2)
KDM1A mRNA (log_2)
D) OHSU AML dataset
Spearman: 0.01
(P = 0.846)
Pearson: 0.01
(P = 0.800)
AXL mRNA (log_2)
KDM1A mRNA (log_2)
E) OHSU AML dataset
Spearman: -0.01
(P = 0.896)
Pearson: -0.13
(P = $6.481e^{-4}$)
MET mRNA (log_2)
KDM1A mRNA (log_2)
F) OHSU AML dataset
Spearman: 0.04
(P = 0.355)
Pearson: 0.03
(P = 0.366)
KITLG mRNA (log_2)
KDM1A mRNA (log_2)

Figure 1

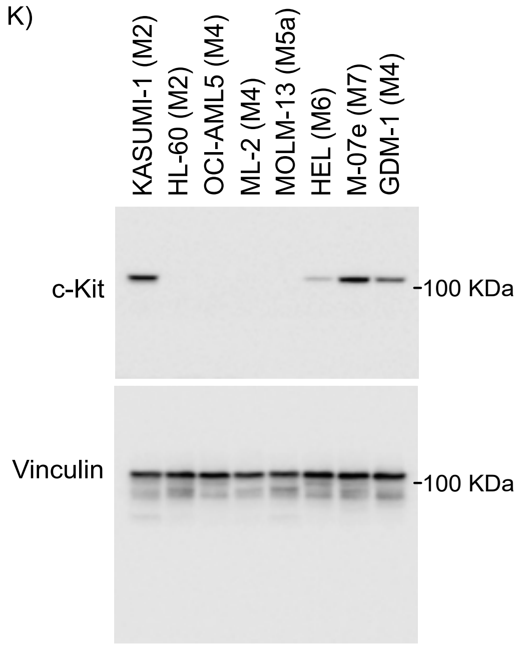

Figure 5

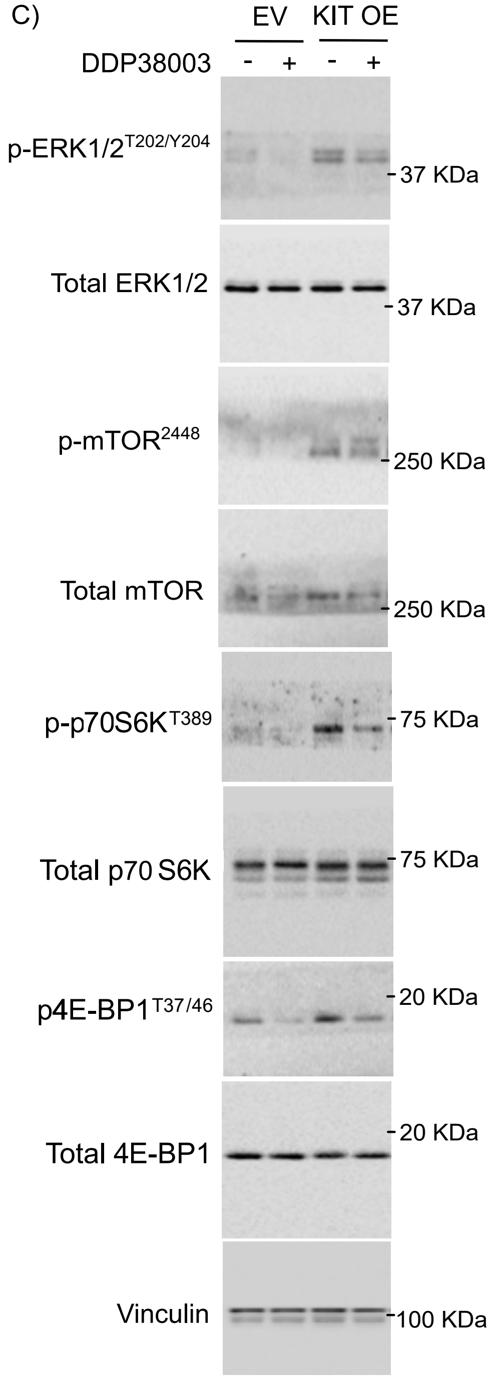

Supplement: Supplementary file 1 — Supplementary Material 1 [file 13148_2026_2098_MOESM1_ESM.pdf]
